# Exploring correlations between neuropsychological measures and domain-specific consistency in associations with n-3 LCPUFA status in 8-9 year-old boys and girls

**DOI:** 10.1371/journal.pone.0216696

**Published:** 2019-05-22

**Authors:** Marie N. Teisen, Janni Niclasen, Stine Vuholm, Jesper Lundbye-Jensen, Ken D. Stark, Camilla T. Damsgaard, Svend S. Geertsen, Lotte Lauritzen

**Affiliations:** 1 Department of Nutrition, Exercise and Sports, Faculty of Science, University of Copenhagen, Copenhagen, Denmark; 2 Steno Diabetes Center Copenhagen, Health Promotion, Diabetes Prevention Research, Copenhagen, Denmark; 3 Frederikshøj Dagbehandlingsskole, Copenhagen, Denmark; 4 Department of Kinesiology, Faculty of Applied Health Sciences, University of Waterloo, Waterloo, Ontario, Canada; Chiba Daigaku, JAPAN

## Abstract

Long-chain n-3 polyunsaturated fatty acids (n-3 LCPUFA) have in some studies been associated with cognitive and socioemotional outcomes in children, but results are inconsistent possibly due to the use of different tests and potential gender-specific effects. The objective of this cross-sectional study was to explore overall patterns in neuropsychological scores as well as correlations between scores within specific domains, and to examine potential gender differences and consistency in associations with n-3 LCPUFA status. In 199 Danish 8–9 year-old children, we performed a large battery of tests and questionnaires on attention, processing speed, executive functions, memory, and socioemotional traits, and measured erythrocyte fatty acid composition. Principal component analyses (PCA) showed that most of the variation in both cognitive performance and socioemotional traits was explained by overall performance, followed by speed-accuracy trade off and externalizing vs. internalizing problems, respectively. Boys had higher speed, lower attention and higher externalizing problem scores than girls. Measures of performance within both processing speed and attention domains correlated moderately, whereas no correlations were found for measures of executive functions apart from some weak correlations for impulsivity. Parent-rated scores for both externalizing and internalizing problems correlated strongly, whereas correlations with child-rated scores were weak. Scores within specific domains did not consistently associate with n-3 LCPUFA, except for processing speed measures which all pointed to faster processing with increased n-3 LCPUFA status. Gender differences in the associations were observed for attention and impulsivity. Child- but not parent-rated internalizing and social problems tended to associate directly with n-3 LCPUFA, supported by increased internalizing problems measured by the PCA component. In conclusion, measures of speed and attention seem to represent these domains in general, whereas single measures of more complex cognitive functions should be interpreted with caution. One approach could be to use multiple tests and create multivariate scores to guide interpretations. Furthermore, the results indicate a need to consider both parent- and child-rated socioemotional scores and gender differences in neuropsychological functions e.g. in investigations of n-3 LCPUFA effects.

## Introduction

Long-chain n-3 polyunsaturated fatty acids (n-3 LCPUFA), mainly docosahexaenoic acid (DHA, 22:6 n-3), are considered important for brain development of children. A considerable amount of DHA is accreted in the brain during perinatal development [1], but accretion continues throughout childhood, especially in the frontal cortex, which is involved in cognitive functions such as attention, memory, and planning [2]. Moreover, DHA has been shown to affect neuronal function and has therefore been hypothesized to affect cognitive function in children [2].

Oily fish is the primary dietary source of n-3 LCPUFA, and many studies have investigated potential associations between intake of n-3 LCPUFA and cognitive performance, behavior and emotional traits [3–7]. Most of the studies have been performed in infants and children with mental disorders such as attention-deficit hyperactivity disorder (ADHD) [8], but in the last decade several randomized clinical trials (RCTs) have examined the effect in healthy schoolchildren [9–20]. Although cross-sectional studies in healthy schoolchildren generally suggest beneficial associations between n-3 LCPUFA intake and neuropsychological functions [21–25], the results of the RCTs are inconsistent. Some RCTs show beneficial effects of n-3 LCPUFA intake on cognitive functions such as processing speed [11, 18, 20], executive functions (especially impulsivity) [13, 18], and memory [10, 15] as well as on socioemotional traits such as externalizing [16, 26], internalizing [11], and social behavior [13], and one study finds increased cortical activation during an attention task [14]. In contrast, others do not find any effects of n-3 LCPUFA on processing speed [12, 19], executive functions [11, 12], memory [9, 11–13, 16, 18], and attention [9, 13, 18], and meta-analyses of RCTs show no effects of n-3 LCPUFA supplements on any cognitive domains in healthy children [6, 7].

A number of different tests and questionnaires have been used to assess association between n-3 LCPUFA status and neuropsychological function in the different studies, and conclusions are typically based on a single test supposed to reflect a certain neuropsychological domain. Differences in methodology could give rise to inconsistent results across studies, and it is unclear to what extent each of the different neuropsychological measures give an overall reflection of a specific neuropsychological domain. Gender differences in the associations may also contribute to the inconsistencies as some studies report gender-specific associations between n-3 LCPUFA and cognitive performance in schoolchildren [15, 23, 28] and status or intake early in life and behavior and social skills later in childhood [29, 30]. The potential modifying effect of gender has so far not been given much attention, and none of the previous studies have examined if associations with n-3 LCPUFA in certain domains depend on the employed measures.

In this study of healthy 8-9-year-old schoolchildren, we performed a large battery of tests frequently used to assess attention, processing speed, memory and executive functions including impulsivity, inhibition and cognitive flexibility as well as questionnaires often used to assess internalizing, externalizing and social problems. We assessed patterns across all tests and questionnaires to derive component scores that could reflect overall cognitive performance and socioemotional behavior. We furthermore explored correlations between scores within the specific domains to assess whether test scores consistently reflected these domains and if some measures of a certain domain appeared to be more sensitive and representative. Additionally, we explored differences between boys and girls in the overall scores and individual domain-specific measures. In order to further investigate consistency in scores within the specific domains, we investigated associations between the overall components as well as measures within the different domains and erythrocyte n-3 LCPUFA in all children and in boys and girls separately.

## Methods

### Study design

This study was an exploration of baseline data from the FiSK Junior trial in which healthy Danish schoolchildren were randomized to receive oily fish or poultry (control) for 12±2 weeks. A comprehensive description of the study design was provided previously [31]. The baseline visits took place between August 2016 and March 2017. The study was conducted in accordance with guidelines in the Declaration of Helsinki and the study protocol (S1 Protocol) was approved by the Committee on Biomedical Research Ethics for the Capital Region of Denmark (H-16018225). Informed written consent was obtained from all custody holders of the children, and the Danish Act on Processing of Personal Data was followed for all data collected in the study. The study is registered in ClinicalTrials.gov (NCT02809508).

### Participants

**Fig 1:**
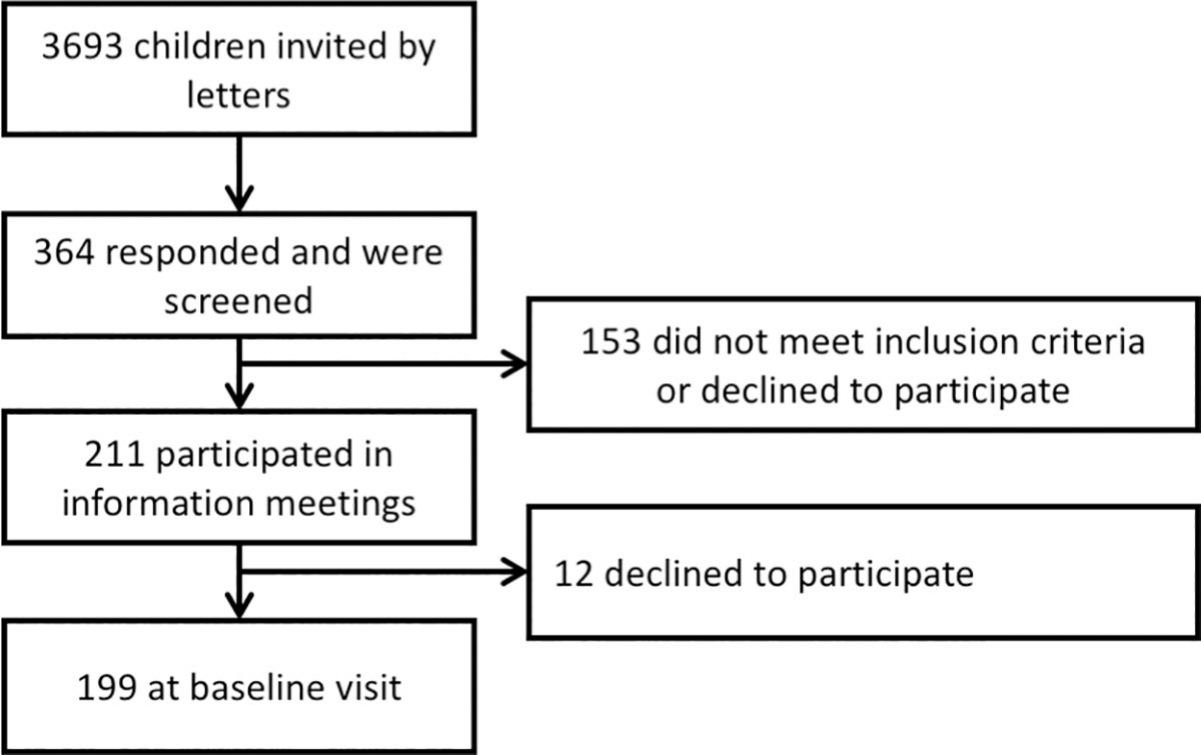
Flowchart of participants.

Children, 8–9 years of age, living in the Capital Region of Denmark were identified through the Danish Civil Registration System and invited by letter to participate in the study. Inclusion criteria were that the child was healthy, spoke Danish, liked oily fish and poultry, did not consume oily fish more than once per week, and did not take any n-3 LCPUFA supplements three months prior to the intervention start. Moreover, parents should read and speak Danish in order to be properly informed about the study procedures. Exclusion criteria were chronic diseases (e.g. diagnosed ADHD or other psychiatric illness), intake of medication that could interfere with the study outcomes, or concomitant participation in other studies involving dietary supplements or blood sampling. In addition, only one child from each household was allowed to participate in the study. Invitations were sent out to 3693 children, 364 responded and were screened, and 211 children participated in information meetings of whom 12 declined to participate, thus leaving a total of 199 children with baseline data (Fig 1).

### Measurements

Cognitive tests, biological sampling and anthropometric measurements were performed by project staff according to standard operating procedures. Questionnaires on sociodemographic background, habitual fish intake and socioemotional problems were answered by one of the parents. The child answered a child version of the KINDL questionnaire of quality of life in an interview with a trained project staff member. Pubertal stage was evaluated by Tanner scores based on questionnaires on breast development and menarche for girls and development of pubic hair for boys, which were answered by the child with aid from a parent. Children’s physical activity was recorded using tri-axial accelerometers (ActiGraph, Pensacola, FL, USA) during 7 consecutive days and 8 nights prior to the examination.

#### n-3 LCPUFA status

Fasting blood was collected by venipuncture, and erythrocytes were isolated by centrifugation and washing three times with saline to remove plasma and leukocytes. The isolated erythrocytes were reconstituted in saline with 2,6-di-tert-butyl-4-methylphenol (butylated hydroxytoluene; Sigma-Aldrich) and stored at -80°C. Within 9 months after blood sampling, erythrocyte fatty acid composition was analyzed by fast gas chromatography at the Department of Kinesiology, University of Waterloo, Canada (ON) as previously described [32]. Lipids were double extracted with a solution of chloroform and methanol containing an internal standard (22:3n-3 ethyl ester, Nu-Chek Prep, Elysian, MN, USA). Fatty acid methyl esters were generated by direct trans-esterification with 14% boron trifluoride in methanol (Sigma- Aldrich, St. Louis, MO, USA) and hexane for 60 minutes at 90°C and subsequently extracted and analyzed on a Varian 3900 gas chromatograph equipped with a DB-FFAP capillary column (15 m × 0·10 mm i.d. × 0·10 μm film thickness; J&W Scientific from Agilent Technologies). The inter- and intra-assay coefficients of variation were below 5% for all identified fatty acids. n-3 LCPUFA status is expressed as eicosapentaenoic acid (EPA, 20:5 n-3) + DHA w/w% of total erythrocyte fatty acids.

#### Cognitive functions

A battery of cognitive tests was selected to obtain measures of performance in the cognitive domains of interest: processing speed, attention, executive functions (impulsivity, inhibition and cognitive flexibility), and memory. The battery consisted of the d2 test of attention, a Stroop task, a modified Flanker task including a switch task part, and four tests from the Cambridge Neuropsychological Automated Battery (CANTAB): Reaction Time (RTI), Rapid Visual Processing (RVP), Paired Associates Learning (PAL), and Spatial Working Memory (SWM). The children were familiarized with the d2 and CANTAB tests in a pre-testing session at the information meetings. Most of the tests provided measures for more of the domains of interest and the measures that we chose to use are shown in Table 1 and described below.

**Table 1:** Overview of domains tested in the different cognitive tests.

Domain/ Task	Processing speed	Attention	Impulsivity	Inhibition	Cognitive flexibility	Memory
**d2 test of attention**	Total number of processed characters (d2t)	Errors of omission % (d2inatt) Total error % (d2te)	Errors of commission % (d2imp)	-	-	-
**Stroop task**	Time on word task (STwt), color task (STct) and color-word task (STit)	-	-	Stroop effect (STeff)	-	-
**Flanker and switch task**	Flanker reaction time (FLrt), Switch reaction time (SWrt)	Flanker total error % (FLte), Errors in congruent trials % (FLcon), Switch accuracy % (SWra)	Errors in incongruent trials % (FLinc)	Flanker effect (FLeff)	Switch cost (SWeff) Mixing cost (MIXeff)	-
**Reaction Time (RTI)**	Median reaction time on 1- (RTIsrt) and 5-choice task (RTIfrt)	SD of mean 5-choice reaction time (RTIfrtsd)	-	-	-	-
**Rapid Visual Processing (RVP)**	-	Misses % (RVPm), Total error % (RVPte)	False alarms % (RVPfa)	-	-	-
**Spatial Working Memory (SWM)**	-	-	-	-	-	Total between errors (SWMbe) Strategy score (SWMs)
**Paired Associates Learning (PAL)**	-	-	-	-	-	Total error % (PALte) Memory score (PALms)

The d2 test of attention provides measures of processing speed, attention and impulsivity. The test-sheet consists of lines of characters (“d’s” and “p’s”) with one to four dashes distributed above and/or below the character. The child was requested to cross out all d’s with two dashes (d2’s) without crossing out distracter characters (all other characters) and was given 20 seconds per line with no pauses between the lines [33]. A measure of processing speed was defined as the total number of characters processed. A measure of impulsivity was defined as commission error % (the number of incorrectly crossed out distractor characters divided by total number of characters processed). Inattention was measured both as omission error % (number of unmarked d2’s divided by total number of characters processed) and total error % (omission error % + commission error %) [34]. The classical scoring of the d2 test also includes a measure of concentration performance (number of correctly crossed out characters minus number of incorrectly crossed out characters), but we did not use this measure as it has been shown to correlate strongly (r = 0.92) with processing speed [33].

The Stroop task assesses inhibition of cognitive interference, which occurs when processing of one feature of a stimulus impedes simultaneous processing of another feature of the same stimulus [35]. This cognitive interference is known as the Stroop effect [36] and the test also provides measures of processing speed. We used a color and word version, where words, colors and colors of words on three separate cards, were read out loud by the child as quickly as possible. If an error was made, this should be corrected before moving on to the next word/color. Measures of processing speed were defined as time on the word, color and color-word subtests and the Stroop effect was calculated as:

$$Stroop\;effect=\frac{colorword\;time-\frac{word\;time+color\;time}{2}}{\frac{word\;time+color\;time}{2}}*Average\frac{word\;time+color\;time}{2}$$

Where *Average (word time + color time)/2* was the average for all children. The numerator in the first part of the equation was previously proposed as a measure of interference [37], but we included the additional adjustment for time on the word and color tests to provide a measure of interference that did not depend on the individual child’s processing speed, and thus allowed comparison of inhibition adjusted for speed across children.

The Flanker task is another test of inhibition [38], which also provides measures of processing speed and attention. We used a modified Flanker task with arrays of fish described in detail in [39]. The child was requested to press the outermost left or right button on a keyboard as quickly and accurately as possible based on the direction of the middle fish (S1A Fig). The direction of the flanking fish was either the same (congruent) or opposite (incongruent) to the middle fish. The used measure of processing speed was reaction time across all trials (Flanker reaction time), and a measure of inhibition, the Flanker effect, which like the Stroop effect was adjusted for speed by the following equation:

$$Flanker\;effect=\frac{Time\;incongruent\;trials-Time\;congruent\;trials}{Time\;congruent\;trials}*Average\;time\;congruent\;trials$$

Where *Average time congruent trials* was the average time across all children in congruent trials. Error % on all trials was used as a measure of attention. The Flanker test does not distinguish errors of omission and commission, but we hypothesized that error % in congruent trials could reflect inattention, and error % in incongruent trials could reflect impulsivity, as it requires impulse inhibition to ignore flanking fish pointing in the wrong direction. A switch task was added to the basic Flanker task to provide a measure of cognitive flexibility. This part included arrays of pink fish in addition to the arrays of blue fish. When the fish were pink, the child had to press based on the direction of the flanking fish, whereas when the fish were blue the child still had to press based on the direction of the middle fish. When the color of fish and thereby the rule changed from the previous trial, a trial was defined as a switch trial, whereas a trial with the same color of fish as the previous trial was defined as a non-switch trial. The main measure used from this task was the ‘switch cost’, which was speed-adjusted by the following equation:

$$Switch\;cost=\frac{Time\;switch\;trials-Time\;nonswitch\;trials}{Time\;nonswitch\;trials}*Average\;time\;nonswitch\;trials$$

Where *Average time nonswitch trials* was average time on non-switch trials across all children. To eliminate the Flanker effect, this measure was only based on congruent trials. The switch task allowed calculation of another measure of cognitive flexibility, the ‘mixing cost’, which reflects the decrease in speed that occurs when the child is required to perform two types of tasks (switch task condition) relative to just one task (the basic Flanker) [40]. The speed-adjusted mixing cost was calculated as:

$$Mixing\;cost=\frac{Switch\;reaction\;time-Flanker\;reaction\;time}{Flanker\;reaction\;time}*Average\;Flanker\;reaction\;time$$

Where *Switch reaction time* was reaction time on all trials in the switch task, and *Average Flanker reaction time* was average reaction time for all children in the Flanker task. The switch reaction time was used as a measure of processing speed, and accuracy % on all trials in the switch task was used as a measure of attention.

The CANTAB tests (http://www.cambridgecognition.com) were performed on an iPad Air placed in front of the child in a holder 16 cm from the edge of the table. The children were familiarized to the iPad with a motor screening task before performance of the actual tests.

RTI measures primarily processing speed. The child was required to hold down a button shown on the screen, and when a yellow spot appeared on the screen let go of the button and press on the screen where the dot appeared (S1B Fig). The test included two subtasks, one with a single possible location of the yellow spot (single choice) and one with five possible locations of the yellow spot (five-choice). As processing speed measures, we used the median reaction time (the time from onset of the stimulus until the child released the button) in correctly performed trials for both the single and the five-choice subtasks. We used the SD of the mean reaction time in the five-choice task as a measure of sustained attention as this reflects variation in how fast the child reacts in each of the trials and thus, presumably their attention to the task. Thus, a higher SD of the mean reaction time indicates lower attention.

RVP is used to assesses sustained visual attention and impulsivity. In the test, digits from 2 to 9 appeared on the screen, and the child was requested to detect a target sequence (3-5-7) and to respond to it by pressing a button on the screen (S1C Fig). The impulsivity measure from this test was false alarm % (number of times the child responded to a non-target sequence divided by total number of non-target sequences), and attention measures were misses % (number of target sequences the child failed to respond to divided by total number of target sequences) and total error % (number of the two types of errors divided by total number of sequences).

SWM assesses working memory based on the ability to retain and manipulate spatial information. In the test, the child had to search for yellow tokens hidden inside boxes, starting with four and increasing to eight boxes (S1D Fig). The child had to remember where a token was previously found, since the token could not be in this box again. The selected measures from this test included total ‘between’ errors, i.e. the total number of times across all trials where the child revisited a box in which a token had previously been found. If the child did not finish the trial with eight boxes due to a limit of 40 box selections, it was given the maximum number of between errors made by the other children on this trial. We also used a strategy score that expressed to what extent the child followed a consistent sequence by beginning all searches with a specific box. The strategy score was obtained by counting the number of times the child began a search with a different box in trials with six and eight boxes [41]. Thus, a low score indicated high use of strategy.

PAL assesses visual short-term memory. Boxes were opened automatically on the screen in a randomized order and displayed patterns, starting with two and increasing to eight patterns in each trial. The patterns were displayed one at a time on the screen, and the child had to touch the box where the pattern was previously shown (S1E Fig). If the child made an error, all the boxes were opened again, and the child was not allowed to proceed to the next trial before the trial was successfully completed. The short-term memory measures used from this test were total error % (number of times the child chose an incorrect box plus an adjustment for the maximum number of errors that could be made in the trials they did not reach, divided by maximum number of trials), and a memory score (number of times the child chose a correct box in the first attempt, calculated across all assessed trials divided by maximum number of first attempts).

#### Socioemotional measures

We used the Strength and Difficulties Questionnaire (SDQ), the KINDL questionnaire, and the Behavior Rating Inventory of Executive function (BRIEF) questionnaire to assess the socioemotional domains of interest: externalizing, internalizing, and social problems.

SDQ measures hyperactivity/inattention, conduct problems, emotional symptoms, peer relationship problems and prosocial behavior over the last 3 months [42]. The emotional and peer subscales were combined in an internalizing subscale, conduct problems and hyperactivity subscales were combined in an externalizing subscale, and all but the prosocial score were summed to generate a total difficulties score. Thus, high scores in this questionnaire indicate difficulties, except for the prosocial score.

BRIEF assesses behavior that reflects children’s executive functions [43]. The Global Executive Function score was comprised of two broad subscales: Behavioral Regulation Index and Metacognition Index. The Behavioral Regulation Index included impulse inhibition, flexibility, and emotional control scales. The Metacognition Index included initiation, working memory, planning, organization of materials, and monitoring scales, of which we only focused on working memory. High scores in this questionnaire indicate behavior related to high executive dysfunction.

KINDL assesses quality of life and includes six subscales; physical well-being, emotional well-being, self-esteem, family, friends and school [44], of which we only focused on the emotional and friends scales as well as the total score, which included all six subscales. High scores in this questionnaire indicate high well-being.

### Statistical analysis

Data is presented as mean ± SD, median [IQR], or percentages as appropriate. The level of significance was set at p<0.05.

Principal component analysis (PCA) was used to derive overall patterns in cognitive test performance and socioemotional traits. The two main components were named according to the measures driving them. Scores for each of the components were generated for all children and used as outcome measures in association analyses along with individual measures from the cognitive tests and socioemotional questionnaires.

Correlations within and between the cognitive and socioemotional domains were explored with overall correlograms, using Spearman’s rank correlations. When two measures of the same domain from the same test were strongly correlated (r≥0.5), we chose only the most normally distributed measure for further analyses. Additional correlation analyses within the specific neuropsychological domains were performed using Pearson correlation or Spearman’s rank correlation as appropriate, and these were used to assess how well measures within the domains correlated and identify potential measures that might best represent performance within the domain by correlations with the other measures.

Associations between n-3 LCPUFA status and the PCA components as well as the individual measures of cognitive performance and socioemotional scores were examined by use of general linear regression models (PCA components, all measures of processing speed, reaction time SD, SWM measures and questionnaire scores), or logistic regression models (error percentages in d2, Flanker and RVP as well as the PAL measures). Non-normally distributed scores were log transformed before linear regression and estimates were back transformed. All models included sex, age, grade, household education level, total physical activity, and month of test. For cognitive scores, models additionally included the order in which the cognitive tests were performed and the tester. Models for the child-rated KINDL scores included interviewer. The underlying assumptions of the general linear models were investigated by visual inspection of residual and normal probability plots. The association analyses were performed for all children as well as for boys and girls separately. The associations between n-3 LCPUFA status and the cognitive and socioemotional measures are presented as ß estimate (95% confidence interval (CI)) from linear models and as odds ratio (OR) (95% CI) from logistic regression models.

Data pre-processing was performed using STATA (StataCorp. 2015. *Stata Statistical Software*: *Release 14*. College Station, TX: StataCorp LP.), PCA was performed using SPSS (IBM Corp. Released 2013. IBM SPSS Statistics for Windows, Version 22.0. Armonk, NY: IBM Corp.), and all additional analyses were performed using R studio (RStudio Team (2016). RStudio: Integrated Development for R. RStudio, Inc., Boston, MA URL http://www.rstudio.com/. Version 1.0.153). The extension packages rcorr and Hmisc were used to make overall correlograms, PerformanceAnalytics was used for within-domain correlation analyses, and lme4 and multcomp were used for logistic regression analysis.

## Results

### Baseline characteristics of the study population

The study population included an equal number of boys and girls with a median age of 9.6 years (Table 2). None of the girls had reached menarche, but 34% of the girls and 13% of the boys had entered puberty. The children came from households with long educations compared to the general Danish population [45], and their mean BMI was slightly below the Danish average [46] (Table 2). Although children were recruited based on a low intake of oily fish, their total fish intake was comparable to that of a representative sample of Danish 4-9-year-old children [47], and their erythrocyte n-3 LCPUFA status (EPA + DHA w/w%) was more than twice as high as European reference values in this age group, whereas arachidonic acid (AA, 20:4,n-6) and linoleic acid (LA, 18:2,n-6) were considerably lower [48, 49].

**Table 2:** Baseline characteristics of the study population^1^.

	All (n = 199)	Girls (n = 99)	Boys (n = 100)
	Mean ± SD or median [IQR]	Mean ± SD or median [IQR]	Mean ± SD or median [IQR]
Age, y	9.6 [9.2; 9.7]	9.3 [9.1; 9.7]	9.6 [9.2; 9.7]
Female:Male ratio, %	49.7:50.3	-	-
2nd:3rd:4th grade ratio, %	17.1:79.4:3.5	23.2:71.7:5.1	11.0:87.0:2.0
Puberty, %			
Have entered puberty^2^	23.6	34.3	13.0
Have had first menstruation	0	0	-
Household education level^3^, %			
Vocational, short academic or less	19.1	18.2	20.0
Bachelor’s degree	23.1	24.2	22.0
Master's degree or higher	57.8	57.6	58.0
BMI, kg/m^2^	16.4 ± 2.0	16.6 ± 2.2	16.2 ± 1.8
Weight status^4^, %			
Underweight	13.6	13.1	14.0
Normal weight	78.9	75.8	82.0
Overweight or obese	7.5	11.1	4.0
Total physical activity, counts/min	402 ± 109	370 ± 106	435 ± 103
Fish intake, g/d	13 [9; 19]	13 [8; 21]	12 [9; 17]
Erythrocyte LCPUFAs^5^, w/w%			
LA (18:2,n-6)	8.93 ± 0.73	8.92 ± 0.66	8.94 ± 0.79
AA (20:4,n-6)	12.69 ± 1.63	12.98 ± 1.62	12.42 ± 1.59
EPA (20:5,n-3)	0.50 [0.41; 0.65]	0.54 [0.44; 0.69]	0.46 [0.39; 0.61]
DHA (22:6,n-3)	4.28 ± 0.94	4.46 ± 0.90	4.11 ± 0.95
EPA + DHA	4.83 ± 1.11	5.04 ± 1.05	4.63 ± 1.13

^1^Data are given as mean ± SD for normally distributed continuous variables, median [IQR] for non-normally distributed continuous variables, or percentages for categorical variables.

^2^ Defined as Tanner stage ≥2.

^3^ Categorized according to the parent/guardian with highest education level.

^4^ Based on age- and gender-specific cut-offs defined to pass through BMI 18.5, 25 and 30 kg/m^2^ at age 18 years.

^5^Erythrocyte fatty acid composition was available for 194 children. LCPUFA, long-chain polyunsaturated fatty acid; LA, linoleic acid; AA, arachidonic acid; EPA, eicosapentaenoic acid; DHA, docosahexaenoic acid.

The children’s cognitive performance in the d2 and CANTAB tests (Table 3) was comparable to Danish children of similar age [19, 50]. No gender differences were observed in the cognitive test measures of executive function. However, boys had lower working memory score (i.e. higher BRIEF working memory), shorter reaction times and lower attention (i.e. more inattention errors) than girls (Tables 3 and 4). Furthermore, boys had higher externalizing problems scores, and also tended to have lower prosocial score compared to girls (p = 0.084) (Table 4). These differences were reflected in a higher SDQ total difficulties score (p = 0.037) and a tendency to higher BRIEF Global Executive Function score (p = 0.086) in boys, indicating better overall executive function in girls compared to boys.

**Table 3:** Baseline scores in cognitive tests^1^.

	All	Girls	Boys	Difference
	Mean ± SD or median [IQR]	Mean ± SD or median [IQR]	Mean ± SD or median [IQR]	p-value^2^
*Processing speed*				
d2 processing speed, characters	303 ± 46	303 ± 48	303 ± 44	0.950
Stroop word time, s	44 ± 7	44 ± 8	44 ± 6	0.960
Stroop color time, s	67 ± 14	65 ± 14	68 ± 14	0.150
Stroop color-word time, s	133 ± 30	131 ± 29	134 ± 31	0.470
Flanker reaction time, ms	721 ± 152	752 ± 176	691 ± 117	0.005
Switch reaction time, ms	1322 ± 216	1380 ± 205	1264 ± 212	<0.001
RTI 1-choice reaction time, ms	366 ± 35	371 ± 37	361 ± 33	0.050
RTI 5-choice reaction time, ms	419 ± 48	432 ± 54	406 ± 39	<0.001
*Attention*				
d2 total error %	1.53 [0.67; 2.85]	1.18 [0.59; 2.72]	1.66 [0.83; 3.05]	0.060
RVP total error %	2.33 [1.67; 3.17]	2.33 [1.67; 2.92]	2.33 [1.67; 3.17]	0.330
Flanker total error %	1.67 [0.0; 3.33]	1.67 [0.00; 3.33]	1.67 [1.67; 3.33]	0.003
Switch accuracy %	92.0 [88.0; 94.0]	93.0 [89.0; 95.0]	91.0 [88.0; 94.0]	0.040
d2 inattention error %	1.18 [0.58; 2.31]	0.86 [0.43; 2.16]	1.59 [0.67; 2.52]	0.040
RVP misses %	16.7 [11.1; 24.1]	16.7 [11.1; 22.2]	16.7 [11.1; 24.1]	0.770
RTI 5-choice reaction time SD, ms	79 ± 53	77 ± 39	81 ± 64	0.560
Flanker congruent error %	0.00 [0.00; 3.33]	0.00 [0.00; 3.33]	0.00 [0.00; 3.33]	0.001
*Impulsivity*				
d2 impulsivity error %	0.00 [0.00; 0.46]	0.00 [0.00; 0.46]	0.00 [0.00; 0.43]	0.700
RVP false alarm %	0.92 [0.55; 1.47]	0.73 [0.55; 1.28]	1.10 [0.55; 1.56]	0.140
Flanker incongruent error %	3.33 [0.00; 3.33]	0.00 [0.00; 3.33]	3.33 [0.00; 5.00]	0.170
*Inhibition*				
Stroop effect, s	78 ± 21	78 ± 22	77 ± 20	0.760
Flanker effect, ms	50 ± 56	50 ± 57	50 ± 55	1.000
*Cognitive flexibility*				
Switch cost, ms	241 ± 152	250 ± 143	232 ± 162	0.430
Mixing cost, ms	628 ± 224	644 ± 245	613 ± 201	0.330
*Working memory*				
SWM total between errors	12 [6; 17]	13 [5.5; 16]	12 [6; 18]	0.700
SWM strategy score	7.9 ± 1.9	8.0 ± 1.8	7.9 ± 2.0	0.850
*Short term memory*				
PAL total error %	8.57 [4.29; 14.29]	7.14 [4.29; 12.86]	8.57 [4.29; 14.29]	0.300
PAL memory score	75 [65; 85]	80 [65; 85]	75 [65; 85]	0.710

^1^Data are given as mean ± SD for normally distributed continuous variables, or median [IQR] for non-normally distributed continuous variables.

^2^Statistical comparisons between boys and girls were made using Student’s T test for normally distributed variables or Mann-Whitney Test for non-normally distributed variables. RTI, Reaction time; RVP, Rapid Visual Processing; SWM, Spatial Working Memory; PAL, Paired Associates Learning.

**Table 4:** Baseline scores in socioemotional questionnaires^1^.

	All	Girls	Boys	Difference
	Mean ± SD or median [IQR]	Mean ± SD or median [IQR]	Mean ± SD or median [IQR]	p-value^2^
*Externalizing problems*				
SDQ externalizing problems	2 [1; 5]	2 [0; 3]	3 [1; 5]	0.001
SDQ conduct problems	0 [0; 1]	0 [0; 1]	0 [0; 1.25]	0.262
SDQ hyperactivity/inattention	1 [0; 4]	1 [0; 3]	3 [0.75; 4]	0.001
BRIEF behavior regulation index	41 ± 9	41 ± 8	41 ± 9	0.627
BRIEF impulsivity	13 [11; 16]	13 [11; 15]	14 [12; 17]	0.043
*Internalizing problems*				
SDQ internalizing problems	2 [1; 4]	2 [1; 4]	2 [0; 4]	0.484
SDQ emotional symptoms	1 [0; 3]	2 [0.5; 3]	1 [0; 3]	0.307
BRIEF emotional control	15 [12; 18]	16 [13; 18]	14 [11; 18]	0.204
KINDLP emotional well-being	88 [78; 94]	88 [78; 94]	88 [80; 94]	0.546
KINDLC emotional well-being	82 ± 13	81 ± 12	82 ± 13	0.387
*Prosocial behavior*				
SDQ prosocial score	9 [8; 10]	10 [9; 10]	9 [8; 10]	0.084
KINDLP friends	81 [75; 94]	81 [75; 94]	81 [75; 94]	0.747
KINDLC friends	81 [75; 94]	81 [75; 94]	81 [75; 89]	0.550
*Total problems*				
SDQ total difficulties	4 [2; 8]	4 [2; 6.5]	6 [3; 9]	0.037
KINDLP total well-being	79 ± 8	78 ± 79	79 ± 8	0.749
KINDLC total well-being	73 ± 10	74 ± 10	74 ± 10	0.983
*Executive functions*				
BRIEF global executive function	111 ± 21	109 ± 19	114 ± 23	0.086
BRIEF flexibility	11 [9; 14]	11 [9; 14]	11 [10; 14]	0.733
BRIEF working memory	14 [12; 17]	14 [12; 16]	15 [12; 18]	0.002

^1^Data are given as mean ± SD for normally distributed variables, or median [IQR] for non-normally distributed variables.

^2^Statistical comparisons between boys and girls were made using Student’s T test for normally distributed variables or Mann-Whitney Test for non-normally distributed variables. SDQ, Strength and Difficulties Questionnaire; BRIEF, Behavior Rating Inventory of Executive Function; KINDLP, parent-rated KINDL; KINDLC, child-rated KINDL

### Overall patterns in cognitive test performance and within-domain correlations

**Fig 2:**
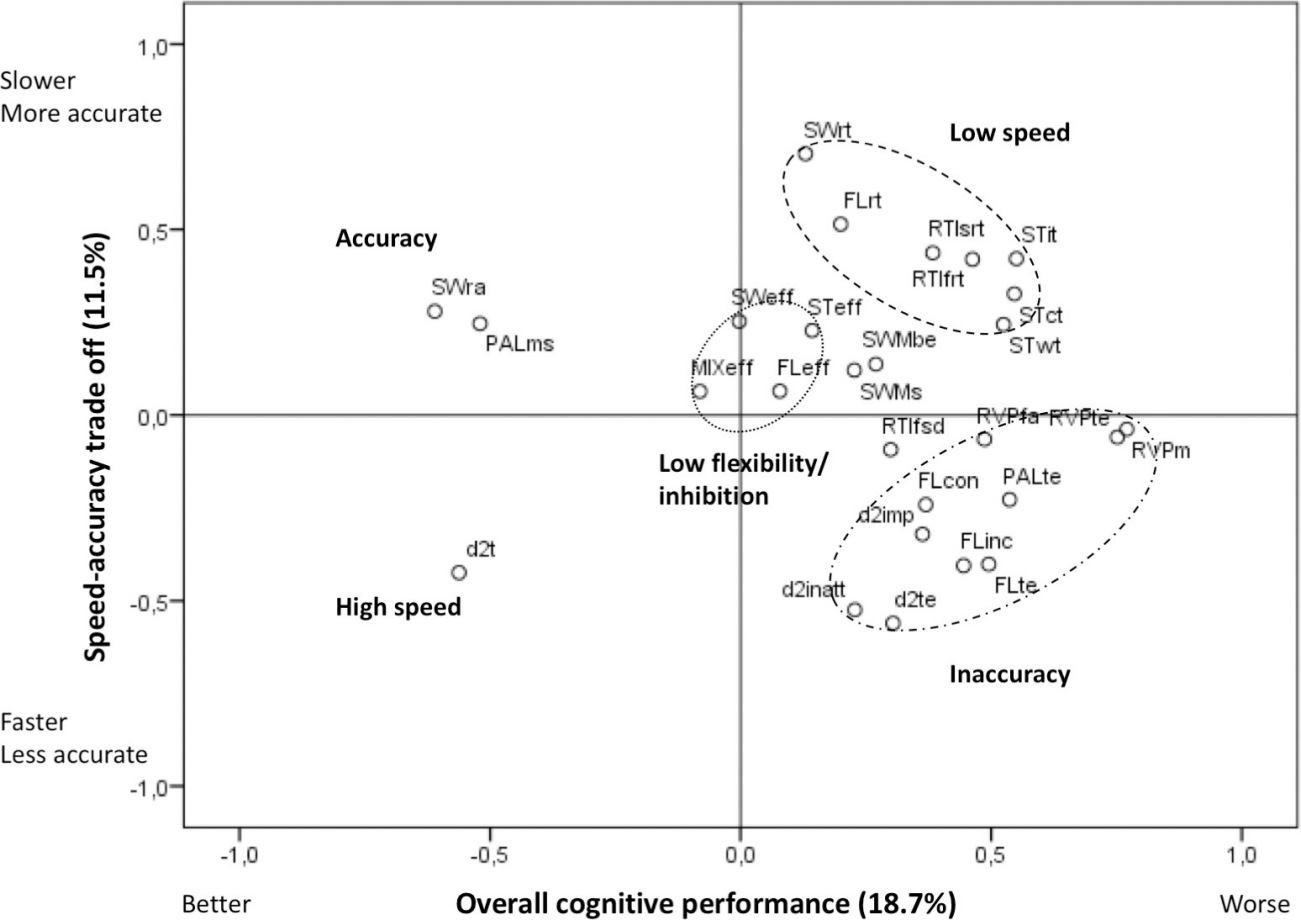
Loading plot from principal component analysis on cognitive test performance. Dashed rings are added to the plot to group outcomes and allow interpretation of the principal components. SWra, switch task response accuracy %; PALms, Paired associates learning memory score; d2t, d2 Processing speed; MIXeff, mixing cost; SWeff, switch cost; FLeff, Flanker effect; STeff, Stroop effect; SWrt, switch task reaction time; STit, Stroop interference time; STct, Stroop color time; STwt, Stroop word time; RTIsrt, simple median reaction time; RTIfrt, five-choice median reaction time; FLrt, Flanker task reaction time; SWMbe, SWM between errors; SWMs, SWM strategy; FLcon, Flanker congruent error %; FLinc, Flanker incongruent error %; FLte, Flanker total error %; RVPte, RVP total error %; RVPm, RVP misses %; RVPfa, RVP false alarm %; PALte, PAL total error %; d2te, d2 total error %; d2inatt, d2 inattention error %; d2imp, d2 impulsivity error %; RTIfsd, five-choice mean reaction time SD.

**Fig 3:**
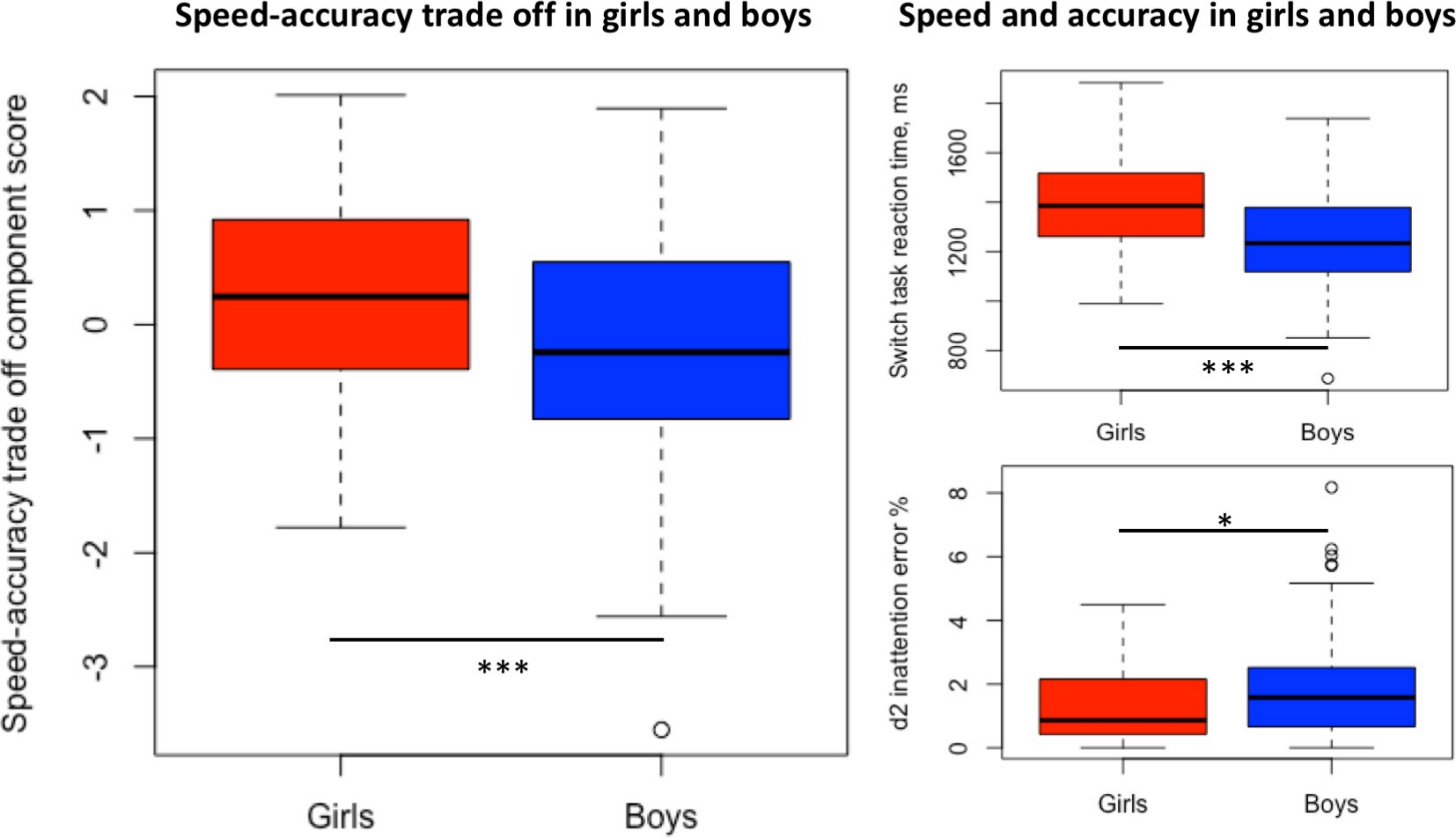
Differences between girls and boys in measures of speed and accuracy. Boxplots of scores in the *Speed-accuracy trade off* component, switch task reaction time and d2 inattention error % in girls and boys. Statistical comparisons between girls and boys were made using Student’s T test for the *Speed-accuracy trade off* component and switch task reaction time and Mann-Whitney Test for d2 inattention error %. ***p<0.001, *p<0.05.

The two principal components in the PCA with all cognitive measures explained 18.7% and 11.5% of the overall variation in test performance (Fig 2). The first component was named *Overall cognitive performance* as it was driven by d2 processing speed and switch task accuracy (negative loadings) and measures of low processing speed and inaccuracy related to both inattention and impulsivity (positive loadings). Thus, a low score on this component reflects good cognitive performance. The second component was mainly driven by high processing speed and inaccuracy in the d2 test (negative loadings) and accuracy and long reaction times in the switch task (positive loadings) and was therefore named *Speed-accuracy trade off*. A low score on this component reflects high speed and low accuracy. There was no significant difference between boys and girls in the *Overall cognitive performance* component, but boys had lower scores in *Speed-accuracy trade off* than girls (ß = -0.51 [-0.79; -0.23], p<0.001), reflecting higher speed and lower accuracy in line with the observed differences in the individual speed and accuracy measures (Fig 3).

A correlogram showed overall correlations within some, but not all domains, and also some correlations across domains (S2 Fig). Due to high correlations between measures of processing speed and attention from the same tests, the ones with the lowest range of variation among the children (i.e. those with a tendency to ceiling or floor effect) were excluded in further analyses. The most consistent correlations between measures from the different tests within a specific domain were seen for processing speed (numeric r in the range 0.22–0.40, p<0.01) (S3 Fig) and attention (r = 0.14–0.47, p<0.05) (S4 Fig). d2 processing speed showed the strongest correlations with measures from the other tests and in the attention domain, accuracy in the switch task was most strongly correlated with the other measures, indicating that these could be good measures of overall function in their respective domains. Weak correlations were observed between impulsivity measures, but all three measures correlated with parent-rated impulse inhibition in the BRIEF questionnaire (r = 0.17–0.21, p<0.05), and thus all seemed to reflect aspects of impulsivity (S5 Fig). No within-domain correlations were observed for the inhibition and cognitive flexibility domains, and none of these measures were correlated with parent-rated flexibility in the BRIEF questionnaire (S6 Fig). Within the memory domain we used the PAL memory score and SWM strategy score which were both strongly correlated with other measures from these tests. These measures did not correlate with each other (S7 Fig), which is in line with their reflection of different memory domains, but the SWM measure did not correlate with the score for working memory from the BRIEF questionnaire (S7 Fig). The PAL memory score was found to correlate with most of the measures of attention (S2 Fig), which indicates that short-term memory depends on attention. Despite our attempt to adjust for speed, the Stroop effect and mixing cost measure were strongly correlated with the processing speed measures that they were based on (S2 Fig).

### Overall patterns in socioemotional problems and within-domain correlations

**Fig 4:**
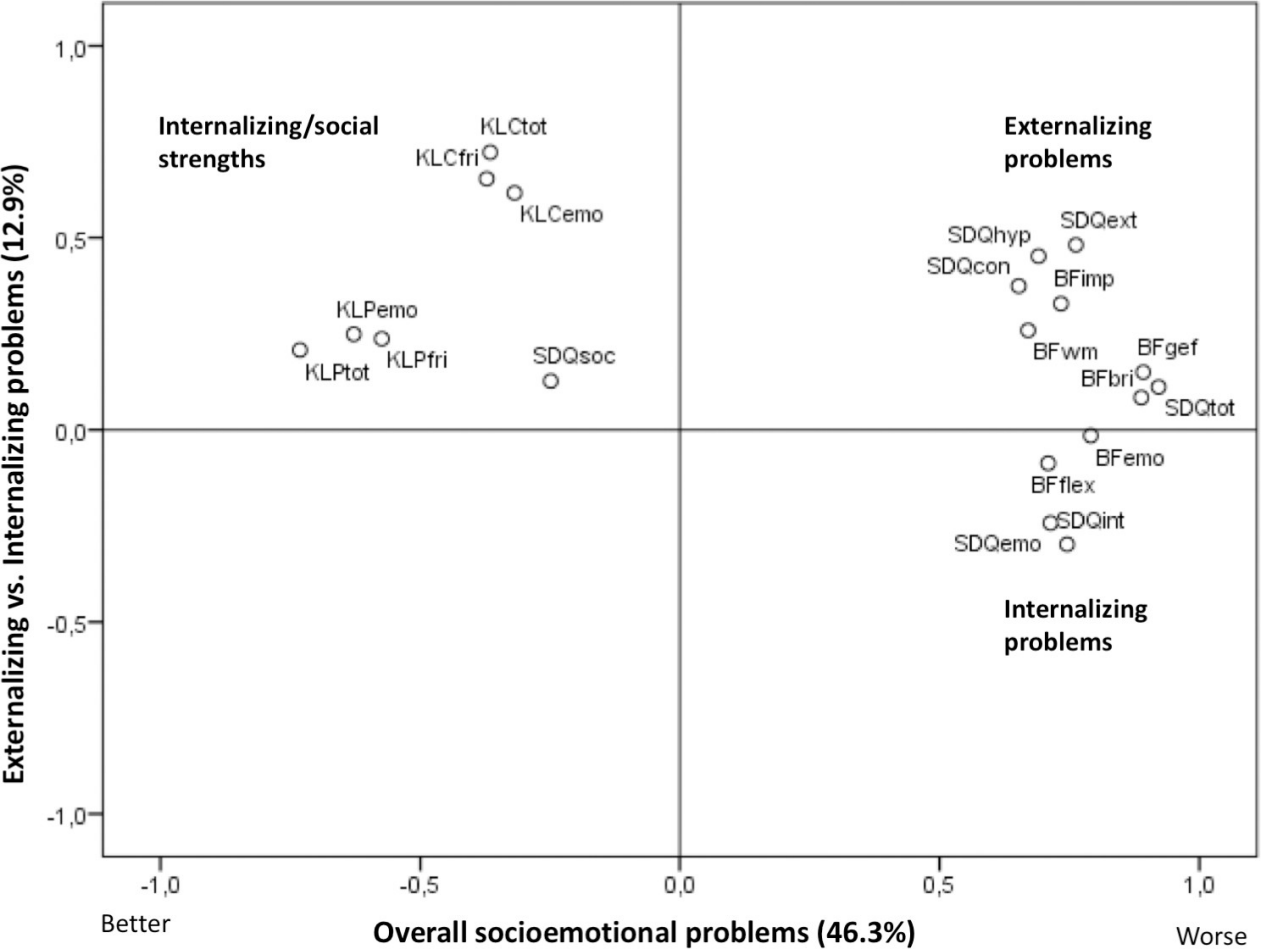
Loading plot from principal component analysis on socioemotional measures. Names added to quadrants in the plot according to the domain mainly represented in each quadrant. BFimp, BRIEF impulsivity; BFflex, BRIEF flexibility; BFgef, BRIEF general executive function; BFwm, BRIEF working memory; BFbri, BRIEF behavior regulation index; SDQcon, SDQ conduct problems; SDQhyp, SDQ hyperactivity/inattention; SDQext, SDQ externalizing problems; KLCemo, KINDL child emotional well-being; KLPemo, KINDL parent emotional well-being; BFemo, BRIEF emotional control; SDQemo, SDQ emotional symptoms; SDQint, SDQ internalizing problems; KLCfri, KINDL child friends; KLPfri, KINDL parent friends; SDQsoc, SDQ prosocial behavior; KLCtot, KINDL child total well-being; KLPtot, KINDL parent total well-being; SDQtot, SDQ total difficulties.

The two principal components derived from the PCA with all the socioemotional measures explained 46.3% and 12.9% of the variation (Fig 4). The first component was named *Overall socioemotional problems* as it was driven by problem scores (positive loadings) and internalizing/social strengths (negative loadings), and a high score on this component therefore indicates more socioemotional problems. The positive loadings for the second component was SDQ externalizing problems, SDQ hyperactivity/inattention, SDQ conduct problems, and BRIEF impulsivity as well as child-rated KINDL emotional well-being and friends scores, and the most negative loading was SDQ internalizing problems. This component was therefore named *Externalizing vs*. *Internalizing problems*, and a high score on this component indicates externalizing as opposed to internalizing problem tendencies. There was no difference between boys and girls in the *Overall socioemotional problems* component, but boys had higher *Externalizing vs*. *Internalizing problems* scores than girls (ß = 0.38 [0.10; 0.66], p = 0.007).

Parent-rated scores were generally correlated across domains (S8 Fig) and the parent-rated total problem scores were highly correlated (r = -0.58, p<0.001), whereas the parent- and child-rated total problem score measured by KINDL were only weakly correlated (r = 0.35, p<0.001) (S9 Fig). Within the externalizing and internalizing domains, scores from the same questionnaires were strongly correlated (S8 Fig), so for externalizing problems we chose to focus only on BRIEF impulsivity and SDQ externalizing problems, and for internalizing problems we excluded the SDQ emotional symptom score in further analyses. The chosen externalizing scores were strongly correlated (S10 Fig). The internalizing problem domain scores were also generally strongly correlated but all correlations between parent-rated measures and the child-rated measure were weak despite the use of the same KINDL emotional well-being score in both parents and children (S11 Fig). The two parent-reported measures within the social problems domain were well correlated, but the child-rated KINDL friends score only correlated with the same parent-rated score (S12 Fig) and all correlations were weaker than for the externalizing and internalizing problem domains.

### Associations between n-3 LCPUFA status and neuropsychological scores

There was no association between n-3 LCPUFA status and the *Overall cognitive performance* component, but in the analysis with all children significant associations were observed between n-3 LCPUFA status and the measures of processing speed (d2 processing speed) and attention (switch response accuracy) that were driving the negative loading of this PCA component and that correlated best with the other measures in these domains (Table 5). High n-3 LCPUFA status was associated with high d2 processing speed (ß = 7.88 [1.14; 14.62], p = 0.022) and, although not significant, all other speed measure estimates also pointed towards higher speed (lower reaction times) with increasing n-3 LCPUFA status. Switch accuracy was inversely associated with n-3 LCPUFA status (OR = 0.95 [0.91; 0.99], p = 0.016) corresponding to a reduction in accuracy of 0.42%-points per 1 FA% increase, but the direction of the estimates for the other attention measures were inconsistent. No association was observed between n-3 LCPUFA status and the *Speed-accuracy trade off* component, although the direction of the association was negative in line with a higher speed and lower accuracy (i.e. lower attention) with increasing n-3 LCPUFA status (Table 5).

**Table 5:** Association between EPA+DHA w/w% and cognitive performance^1^.

	All		Girls		Boys	
	Estimate^2^ ß or *OR (95% CI)*	EPA+DHA p-value	Estimate^2^ ß or *OR (95% CI)*	EPA+DHA p-value	Estimate^2^ ß or *OR (95% CI)*	EPA+DHA p-value
*Overall cognitive performance*	-0.05 (-0.22; 0.20)	0.504	-0.05 (-0.27; 0.18)	0.673	-0.01 (-0.22; 0.20)	0.911
*Speed-accuracy trade off*	-0.09 (-0.23; 0.05)	0.198	-0.08 (-0.30; 0.13)	0.441	-0.12 (-0.32; 0.08)	0.238
*Processing speed*						
d2 processing speed, characters^a^	7.88 (1.14; 14.62)	0.022	9.84 (-1.07; 20.76)	0.076	6.11 (-3.53; 15.76)	0.211
Stroop color time, s^a^	-0.85 (-2.96; 1.26)	0.427	-2.13 (-5.30; 1.04)	0.184	-0.14 (-3.13; 2.86)	0.929
Switch reaction time, ms^a^	-16.7 (-47.9; 14.5)	0.291	-18.9 (-65.3; 27.5)	0.419	-26.9 (-71.1; 17.2)	0.228
RTI 5-choice reaction time md, ms^a^	-0.35 (-6.79; 6.08)	0.914	1.57 (-10.87; 14.00)	0.802	-5.84 (-13.11; 1.42)	0.113
*Attention*						
Switch accuracy %^b^	*0*.*95 (0*.*91; 0*.*99)*	0.016	*1*.*01 (0*.*94; 1*.*08)*	0.841	*0*.*91 (0*.*86; 0*.*96)*	0.001
d2 inattention error %^b^	*0*.*97 (0*.*91; 1*.*04)*	0.412	*1*.*00 (0*.*90; 1*.*11)*	0.978	*0*.*97 (0*.*88; 1*.*06)*	0.504
RVP misses %^b^	*0*.*98 (0*.*93; 1*.*04)*	0.542	*1*.*06 (0*.*97; 1*.*16)*	0.170	*0*.*93 (0*.*86; 1*.*01)*	0.085
Flanker total error %^b^	*0*.*98 (0*.*87; 1*.*11)*	0.768	*0*.*98 (0*.*80; 1*.*21)*	0.871	*0*.*95 (0*.*81; 1*.*12)*	0.560
RTI 5-choice reaction time SD, ms^a^	0.42 (-7.50; 8.35)	0.916	-2.98 (-12.71; 6.75)	0.543	-0.98 (-14.58; 12.62)	0.886
*Impulsivity*						
d2 impulsivity error %^b^	*0*.*97 (0*.*83; 1*.*12)*	0.636	*0*.*80 (0*.*63; 1*.*01)*	0.057	*1*.*07 (0*.*88; 1*.*30)*	0.520
RVP false alarm %^b^	*1*.*00 (0*.*94; 1*.*07)*	0.889	*0*.*91 (0*.*82; 1*.*01)*	0.072	*1*.*10 (1*.*01; 1*.*21)*	0.026
Flanker incongruent error %^b^	*1*.*00 (0*.*96; 1*.*04)*	0.930	*1*.*00 (0*.*94; 1*.*06)*	0.981	*0*.*99 (0*.*94; 1*.*05)*	0.842
*Inhibition*						
Stroop effect, s^a^	1.85 (-1.46; 5.16)	0.272	-0.95 (-6.21; 4.31)	0.719	3.94 (-0.77; 8.65)	0.099
Flanker effect, ms^a^	2.03 (-6.22; 10.28)	0.628	7.64 (-5.48; 20.75)	0.249	0.10 (-11.64; 11.83)	0.987
*Cognitive flexibility*						
Switch cost, ms^a^	-6.6 (-29.8; 16.6)	0.576	25.8 (-6.5; 58.0)	0.115	-29.7 (-63.6; 4.1)	0.084
Mixing cost, ms^a^	0.1 (-33.0; 33.2)	0.995	-1.2 (-57.9; 55.6)	0.968	-6.4 (-49.0; 36.3)	0.767
*Working memory*						
SWM strategy score^a^	-0.23 (-0.50; 0.04)	0.100	-0.13 (-0.53; 0.28)	0.538	-0.36 (-0.79; 0.07)	0.100
*Short term memory*						
PAL memory score^b^	*0*.*99 (0*.*92; 1*.*07)*	0.852	*0*.*98 (0*.*86; 1*.*11)*	0.737	*0*.*97 (0*.*86; 1*.*09)*	0.609

^1^The association was estimated using ^a^linear regression or ^b^logistic regression. The analysis included sex, age, grade, household education level, total physical activity, month of test, order of cognitive test, and cognitive tester.

^2^Estimates presented as ß (95% CI) for linear models or *odds ratio (95% CI)* given in italic for logistic regression models. RTI, Reaction time; RVP, Rapid Visual Processing; SWM, Spatial Working Memory; PAL, Paired Associates Learning.

Gender-specific associations were indicated for some of the speed and attention measures. The positive association between n-3 LCPUFA and d2 processing speed in all children was reflected in a tendency to positive association in girls whereas the overall negative association with switch accuracy was driven by decreased accuracy only in boys (OR = 0.91 [0.86; 0.96], p = 0.001). In contrast, attention measured by RVP showed that boys made less errors, whereas girls made more with increasing n-3 LCPUFA status (RVP misses % OR = 0.93 [0.86; 1.01] and 1.06 [0.97; 1.16], respectively, p_sex-interaction_ = 0.019). No gender-specific associations were observed for the other attention measures. Although no overall associations were observed for the impulsivity measures, some gender-specific associations were also indicated for this domain as n-3 LCPUFA status associated with increased impulsivity in boys (RVP false alarm % OR = 1.10 [1.01; 1.21], p = 0.026), which tended to decrease in girls (RVP false alarm % OR = 0.91 [0.82; 1.01], p = 0.072 and d2 impulsivity error % OR = 0.80 [0.63; 1.01], p = 0.057). The association between n-3 LCPUFA and switch cost was also dependent on gender (p_sex-interaction_ = 0.012) with a direct association (i.e. lower cognitive flexibility) in girls (ß = 25.8 [-6.5; 58.0], p = 0.115) and an inverse association (i.e. higher cognitive flexibility) in boys (ß = -29.7 [-63.6; 4.1], p = 0.084).

There were no significant overall or gender-specific associations between n-3 LCPUFA status and any of the socioemotional scores (Table 6). No trends were observed for any of the externalizing problems scores, but a tendency was observed for an association between n-3 LCPUFA status and the *Externalizing vs*. *Internalizing problems* component, which indicated more internalizing as opposed to externalizing problems with increasing n-3 LCPUFA status (ß = -0.13 [-0.27; 0.01], p = 0.069). In line with this, child-rated emotional and friends scores tended to be negatively associated with n-3 LCPUFA status (ß = -1.64 [-3.52; 0.24], p = 0.088 and -1.93 [-4.22; 0.35], p = 0.097, respectively), which was reflected in a tendency to a negative association between n-3 LCPUFA status and child-rated total well-being (ß = -1.38 [-2.83; 0.07], p = 0.062). All of these tendencies were driven by borderline significant associations in boys (Table 6), indicating decreased well-being with increased n-3 LCPUFA status mainly in boys. The parent-rated friends score indicated the opposite gender-specific association with n-3 LCPUFA status, as a tendency in a negative direction was observed in girls (ß = -2.75 [-5.80; 0.29], p = 0.076), whereas the direction of the association was positive in boys (ß = 1.68 [-1.63; 4.99], p = 0.316). Despite the observed correlations between the measures within internalizing problems and prosocial domains, none of the other scores within these domains showed any associations with n-3 LCPUFA status overall or in boys and girls separately.

**Table 6:** Association between EPA+DHA w/w% and socioemotional outcomes^1^.

	All		Girls		Boys	
	Estimate^2^ ß (95% CI)	EPA+DHA p-value	Estimate^2^ ß (95% CI)	EPA+DHA p-value	Estimate^2^ ß (95% CI)	EPA+DHA p-value
*Overall socioemotional problems*	0.05 (-0.09; 0.19)	0.521	0.08 (-0.13; 0.28)	0.464	0.00 (-0.21; 0.22)	0.963
*Externalizing vs*. *Internalizing problems*	-0.13 (-0.27; 0.01)	0.069	-0.02 (-0.23; 0.18)	0.809	-0.19 (-0.39; 0.02)	0.072
*Externalizing problems*						
SDQ externalizing problems	-0.11 (-0.51; 0.29)	0.592	0.09 (-0.45; 0.63)	0.740	-0.25 (-0.89; 0.39)	0.443
BRIEF impulsivity	0.06 (-0.36; 0.48)	0.780	0.14 (-0.43; 0.70)	0.629	0.11 (-0.55; 0.77)	0.736
*Internalizing problems*						
SDQ internalizing problems	0.07 (-0.34; 0.48)	0.752	0.16 (-0.51; 0.82)	0.638	-0.12 (-0.65; 0.42)	0.670
BRIEF emotional control	0.11 (-0.45; 0.68)	0.688	0.37 (-0.48;1.22)	0.388	-0.12 (-0.92; 0.69)	0.774
KINDLP emotional well-being	0.11 (-1.86; 2.08)	0.913	1.30 (-1.36; 3.95)	0.334	-0.62 (-3.70; 2.47)	0.693
KINDLC emotional well-being	-1.64 (-3.52; 0.24)	0.088	-0.41 (-3.42; 2.61)	0.788	-3.11 (-6.21; -0.01)	0.050
*Prosocial behavior*						
SDQ prosocial score	-0.09 (-0.30;0.11)	0.366	-0.09 (-0.42; 0.25)	0.614	-0.09 (-0.35; 0.17)	0.480
KINDLP friends	-0.74 (-2.92; 1.44)	0.505	-2.75 (-5.80; 0.29)	0.076	1.68 (-1.63; 4.99)	0.316
KINDLC friends	-1.93 (-4.22; 0.35)	0.097	-0.15 (-0.71; 0.42)	0.609	-0.44 (-0.94; 0.06)	0.086
*Total problems*						
SDQ total difficulties	-0.04 (-0.71; 0.63)	0.899	0.25 (-0.82; 1.31)	0.646	-0.36 (-1.29; 0.56)	0.437
KINDLP total well-being	-0.45 (-1.59; 0.69)	0.434	-0.89 (-2.58; 0.80)	0.296	0.30 (-1.44; 2.05)	0.730
KINDLC total well-being	-1.38 (-2.83; 0.07)	0.062	-0.97 (-3.30; 1.37)	0.414	-2.27 (-4.60; 0.05)	0.055
*Executive functions*						
BRIEF global executive function	1.01 (-1.87; 3.89)	0.489	1.73 (-2.23; 5.68)	0.388	0.42 (-4.13; 4.98)	0.855
BRIEF flexibility	0.16 (-0.25; 0.57)	0.444	0.02 (-0.61; 0.66)	0.939	0.25 (-0.32; 0.81)	0.390
BRIEF working memory	0.23 (-0.28; 0.73)	0.372	0.30 (-0.33; 0.94)	0.348	0.17 (-0.69; 1.02)	0.698

^1^The association was estimated using linear regression. The analysis included sex, age, grade, household education level, total physical activity, month of test, and for KINDLC outcomes additionally interviewer.

^2^Estimates presented as ß (95% CI) and back transformed ß (95% CI) for log transformed variables. SDQ, Strength and Difficulties Questionnaire; BRIEF, Behavior Rating Inventory of Executive Function; KINDLP, parent-rated KINDL; KINDLC, child-rated KINDL.

## Discussion

In this study we found that measures of processing speed, attention and also to some degree impulsivity correlated across tests, whereas measures of inhibition, cognitive flexibility and memory showed no correlations across tests. Moreover, the correlations between parent-rated impulsivity and child performance in tests within these domains were in accordance, whereas those for measures of cognitive flexibility and working memory were not. However, we also observed several correlations across domains underlining that most cognitive tests are influenced by function in more than one cognitive domain. Scores supposed to reflect short-term memory correlated with attention measures suggesting that short-term memory is influenced by attention skills. Furthermore, we observed strong correlations between the traditional inhibition measures in the Flanker and Stroop tasks and reaction time. This prompted us to make speed-adjustments of these measures, but some correlation with speed was still apparent for the adjusted scores, which therefore cannot be interpreted as pure measures of inhibition. Overall, the results underline that memory and executive function domains are highly complex and therefore difficult to assess by performance in a single cognitive test, whereas processing speed and attention measures from single tests seem more generalizable. This is in line with previously reported correlation between the speed and accuracy measures from Flanker and Stroop tasks and lack of correlation between their inhibition measures [51]. A single high-quality test with good reliability and validity might give an indication of a specific aspect of a cognitive function, but if one wants to explore overall performance in a complex cognitive domain, it might be optimal to use more tests and create multivariate component scores. The components that explained most of the variation in our PCA on cognitive scores were related to overall cognitive performance and secondly speed-accuracy trade off, which is in accordance with results from our previous study with a similar approach [28]. The PCA components were driven by measures of processing speed, attention and impulsivity and results for these components were consistent with those of individual measures in the domains, especially with respect to the differences between boys and girls. This underlines the usefulness of such multivariate components to guide overall interpretations.

The PCA on socioemotional scores showed that variation primarily reflects overall socioemotional problems and secondly differences in the nature of the problems as all traits that characterize externalizing problems clustered in one end of the scale and those related to internalizing problems in the other. The parent-rated measures of total as well as internalizing, externalizing, and social problems correlated well across questionnaires. This is in line with previous studies in healthy children showing that parent-rated scores on specific subscales of the SDQ correlated with BRIEF measures of similar domains [52] and that the SDQ total difficulties score predicted total well-being on KINDL [53]. The observed weak correlations between the child- and parent-rated KINDL measures are also in accordance with other studies that observed only weak to moderate correlations between child- and parent-rated scores in KINDL [53] and in other questionnaires [54, 55]. The weak correlations between the scores of parents and children could be due to differences in the children’s perception of the traits and their behavioral manifestation. Many epidemiological studies, for example of early exposures and later well-being of children, use parent-rated scales due to low reliability in scores rated by younger children. However, the strong correlations in parent scores might reflect a general attitude of parents towards their child. One could therefore speculate that parent-rated scores could have low sensitivity towards subtle changes in child well-being. On the other hand, socioemotional traits rated by children may likely depend on their current state of mind, which may increase day-to-day variation. The best compromise may therefore be to use ratings of both children and parents in studies that aim to assess short-term effects on socioemotional traits e.g. in an RCT with n-3 LCPUFA.

To further examine consistencies in the employed cognitive and socioemotional measures, we tested their association with n-3 LCPUFA status. The aim of this study was not to focus on single significant results, but rather to explore whether associations with measures within the different cognitive and socioemotional domains were in accordance and supported by associations with the overall components. There was no association between n-3 LCPUFA and the overall cognitive components from the PCA, but all individual estimates pointed towards higher processing speed with increased n-3 LCPUFA status whereas individual measures of attention did not associate consistently with n-3 LCPUFA, despite their internal correlation. Similarly, no associations were found between n-3 LCPUFA status and the overall socioemotional components although associations were indicated for some of the emotional and social well-being measures.

The only significant association between n-3 LCPUFA and processing speed was with the measure from the d2 test. The differences in strength of association could be due to differences in the sensitivity of the measures. The d2 measure had a wide range of variation between the children, whereas the response times for the Stroop color card and RTI were both skewed towards the left, as most of the children did fairly well in these tests, which would likely result in a low ability to differentiate between the children. Furthermore, in the Stroop task the children were not allowed to continue their reading of the card without correcting their errors, so the Stroop color card processing speed measure depended on the number of errors and is therefore likely also affected by attention. The observed positive association between n-3 LCPUFA status and processing speed are supported by some other studies in children and adolescents [11, 18, 27, 28], but not by other studies [11, 12, 19].

n-3 LCPUFA status was associated with lower accuracy in the switch task, whereas the estimates for several of the other tasks pointed towards a higher attention. We have previously found an association between n-3 LCPUFA and low attention in an RCT with fish containing school meals [19, 28], but three other n-3 LCPUFA RCTs did not find any effects on attention performance [9, 14, 18] and an RCT that only included boys found increased prefrontal cortex activation during an attention task after n-3 LCPUFA supplementation [14]. These inconsistencies could be due to gender differences in the associations with attention as a Danish cross-sectional study showed that increasing n-3 LCPUFA status was associated with increased attention in boys but decreased attention in girls [28]. In line with this, we observed an association between high n-3 LCPUFA status and an increased number of errors in the RVP task in girls and fewer errors in boys, but we also found a decrease in accuracy in the switch task in boys. This could reflect differences between tests e.g. with respect to influences from the speed domain.

Girls and boys differed significantly in the *Speed-accuracy trade off* component from the PCA analysis. This was also reflected in differences between boys and girls in the individual speed and attention measures, where boys had faster processing speed and lower attention than girls. Faster speed and lower attention in boys compared to girls is supported by large studies in this age group [56, 57]. Boys generally had higher externalizing problems scores, which was reflected in a higher *Externalizing vs*. *Internalizing problems* component score than girls. Boys have in line with this been reported to be more impulsive [58] and have a higher prevalence of externalizing disorders such as ADHD and disruptive behavior disorders compared to girls [59, 60], whereas attention disorder typically presents in a more internalizing way in girls [61], who also have a higher prevalence of disorders such as depression and anxiety [62, 63]. These basic gender differences are probably due to brain sex differences, including genetic and hormonal differences that affect biochemical processes and influence behavior differently in boys and girls. These differences might also explain why n-3 LCPUFA exposure could affect cognitive function differently in boys and girls. It has been speculated that girls have a greater need for DHA, because they accrete more peripheral fat stores [64] and a potential positive effect on attention specifically in boys could be due to a higher proportion of the DHA being accreted in the brain. On the other hand, it has also been suggested that boys have a greater need for DHA due to lower endogenous DHA synthesis [65]. A more pronounced effect of n-3 LCPUFA in boys may also reflect the lower baseline attention in boys which could make room for larger improvements. Our results also raise the possibility that differences in effects could be related to differences in the speed-accuracy trade off in boys and girls.

We found some gender differences in associations between executive functions and n-3 LCPUFA status, most pronounced for the switch cost measure, which is supposed to reflect cognitive flexibility. The interpretation of this measure is however questioned by the lack of correlation with other executive function measures in this study. The results from the switch task indicated that girls were more easily distracted with increasing n-3 LCPUFA status, whereas boys were less distracted. Girls tended to be less impulsive with increased n-3 LCPUFA status in both the d2 and RVP tests, whereas boys became more impulsive, especially in the RVP test. It could be speculated that the general higher impulsivity in boys [58] make them less sensitive to distractions and that they would respond to the increased complexity of the switch task with an increase in errors instead of a slower reaction time. This is consistent with the observed decreased switch task accuracy in the in boys with increasing n-3 LCPUFA status and in line with results from a Danish RCT, which showed that d2 impulsivity increased in boys after intervention with fish-rich school meal while it decreased in girls [66]. However, a cross-sectional analysis of baseline data from the same RCT showed that boys were less impulsive with increasing n-3 LCPUFA status [28]. We did not observe any gender-specific associations between parent-rated impulsivity and n-3 LCPUFA status although this could have been expected as parent-rated impulsivity was correlated with the children’s test performance in all the tests with impulsivity measures. None of the parent-rated scores for cognitive flexibility, inhibition, and memory correlated with the children’s test scores in these domains, which could indicate differences between children’s performance on specific tests and their general behavior related to these domains. It could also be due to subjective ratings by parents, which would likely be less sensitive to changes e.g. in response to the child’s n-3 LCPUFA intake.

Inconsistencies in the associations between socioemotional scores and n-3 LCPUFA status might also be explained by differences between child- and parent-rated scores as tendencies of associations between n-3 LCPUFA status and the socioemotional measures were only indicated for the child-rated scores. There was a tendency towards decreased child-rated total well-being with increased n-3 LCPUFA status, which was driven by decreases in internalizing and social strength scores. Considering the lack of associations with other emotional or social scores this could be chance findings but might also reflect subtle differences only perceived by the children. However, there was also a tendency towards an association with the *Externalizing vs*. *Internalizing problems* PCA component indicating a shift from externalizing to internalizing problems with increasing n-3 LCPUFA. The shift towards internalizing problems was driven by boys as they showed borderline significant associations between n-3 LCPUFA status and child-rated total and emotional well-being and friend scores. In contrast, n-3 LCPUFA status associated with higher parent-rated friends score in boys, but lower score in girls. Several studies in healthy schoolchildren have found n-3 LCPUFA associations with socioemotional traits rated by parents or teachers which generally show beneficial associations with increases in prosocial scores [13], and decreases in externalizing and internalizing problem scores [16, 22, 26, 67] but one study also showed increase in teacher-rated SDQ total difficulties [13]. However, results in the present study suggest that parent-rated scores could lack some sensitivity and objectivity, and thus suggest a need to also consider child-rated scores in future studies.

One of the major strengths of this study includes the use of a wide range of tests and questionnaires that have often been employed to assess specific aspects of cognitive and socioemotional function. The observed correlations between measures, which are supposed to assess function in the same cognitive and socioemotional domains strengthens interpretation of results. Furthermore, we added PCA components based on all the cognitive and socioemotional measures, which allowed us to evaluate overall patterns in performance in addition to performance within specific domains. The children in this study were recruited from households in the capital region of Denmark with education levels above average for the general Danish population. However, their performance in cognitive tests was comparable to children with diverse socioeconomically background recruited from several Danish municipal schools [19, 50]. The sample size in this study was determined by power calculations for health outcomes in the subsequent intervention trial (FiSK Junior) [31], and it is a small study when it comes to the analyses of associations with n-3 LCPUFA status. However, the number of children is considered appropriate to assess correlations between test and questionnaire measures, which was the primary aim of the study. Large variations in both the cognitive and socioemotional measures and n-3 LCPUFA status gave our study broad ranges for the correlation and association analyses. In addition, the used objective measure of n-3 LCPUFA status reduces the risk of misclassification as it is more reliable than data obtained by food frequency questionnaires which are often used in large cross-sectional analyses. However, as in all observational studies there is a risk of confounding from other dietary components and lifestyle factors. We did not make adjustments for multiple testing, because the analyses of association with n-3 LCPUFA were mainly used to explore consistencies across test measures. Furthermore, a traditional Bonferroni correction would overcorrect due to correlations between many of the neuropsychological measures. The low power might contribute to the inconsistent associations between n-3 LCPUFA and measures within the same cognitive domains and the borderline associations between n-3 LCPUFA status and socioemotional traits. Future studies with sufficient power are needed to further investigate consistency in the associations between measures within the same neuropsychological domains and n-3 LCPUFA status to give a better understanding of how n-3 LCPUFA affect brain function.

## Conclusions

Our results indicated that measures of speed and attention are somewhat generalizable, whereas no correlations were observed between measures of executive functions underlining the need to use more tests to make good inference about child performance within complex cognitive domains. Correlations between parent- and child-rated socioemotional scores were poor, and gender differences were observed for speed, attention and externalizing problems as well as in associations with n-3 LCPUFA status. We therefore suggest that future studies use both parent- and child-rated questionnaires of neuropsychological function and focus on potential gender differences. We furthermore suggest that future studies of cognitive function use a large battery of tests to evaluate consistency in results and enable generation of multivariate scores to strengthen interpretation of overall function.

## Supporting information

S1 Protocol Study protocol for the FiSK Junior (The effect of fatty fish and poultry on children’s cardiometabolic health and cognitive function) trial approved by the Committee on Biomedical Research Ethics for the Capital Region of Denmark (H-16018225). (PDF)

S1 Fig Flanker and CANTAB tests. A. Flanker task. B. Reaction time (RTI). C. Rapid Visual Processing (RVP). D. Spatial Working Memory (SWM). E. Paired Associates Learning (PAL).
(TIFF)

S2 Fig Bivariate correlations between outcomes from cognitive tests. Blue circles show positive correlations, red circles show negative correlations. Color intensity and size of the circle indicates strength of the association. Blank fields indicate insignificant (p>0.05) correlation. SWra, switch task response accuracy %; PALms, Paired associates learning memory score; d2t, d2 Processing speed; MIXeff, mixing cost; SWeff, switch effect; FLeff, Flanker effect; STeff, Stroop effect; SWrt, switch task reaction time; STit, Stroop color-word time; STct, Stroop color time; STwt, Stroop word time; RTIsrt, simple reaction time; RTIfrt, five-choice reaction time; FLrt, Flanker reaction time; SWMbe, SWM total between errors; SWMs, SWM strategy; FLcon, Flanker congruent error%; FLinc, Flanker incongruent error%; FLte, Flanker total error%; RVPte, RVP total error%; RVPm, RVP misses%; RVPfa, RVP false alarm%; PALte, PAL total error%; d2te, d2 total error%; d2inatt, d2 inattention error%; d2imp, d2 impulsivity error%; RTIfsd, five-choice reaction time SD.
(TIFF)

S3 Fig Processing speed correlations. Numbers indicate Pearson correlations. *p<0.05, **p<0.01, ***p<0.001. d2t, d2 Processing speed; SWrt, switch task reaction time; STct, Stroop color time; RTIfrt, Reaction time five-choice reaction time.
(TIFF)

S4 Fig Attention correlations. Numbers indicate Spearman correlations. *p<0.05, **p<0.01, ***p<0.001. SWra, switch task response accuracy %; RVPm, RVP misses %; d2inatt, d2 inattention error%; RTIfsd, Reaction time five-choice reaction time SD; FLte, Flanker total error %. One d2inatt outlier (>15) and two RTIfsd outliers (>300) removed from the analysis.
(TIFF)

S5 Fig Impulsivity correlations. Numbers indicate Spearman correlations. ^•^p<0.1, *p<0.05, **p<0.01. d2imp, d2 impulsivity error%; RVPfa, RVP false alarm%; FLinc, Flanker incongruent error %; BFimp, BRIEF impulsivity.
(TIFF)

S6 Fig Inhibition and cognitive flexibility correlations. Numbers indicate Spearman correlations. *p<0.05. STeff, Stroop effect; FLeff, Flanker effect; SWeff, switch cost; MIXeff, mixing cost; BFflex, BRIEF flexibility.
(TIFF)

S7 Fig Memory correlations. Numbers indicate Spearman correlations. *p<0.05, **p<0.01, ***p<0.001. PALms, Paired associates learning memory score; SWMs, SWM strategy; BFwm, BRIEF working memory.
(TIFF)

S8 Fig Bivariate correlations between outcomes from questionnaires. Blue circles show positive correlations, red circles show negative correlations. Color intensity and size of the circle indicates strength of the association. Blank fields indicate insignificant (p>0.05) correlation. BFgef, BRIEF general executive function; BFwm, BRIEF working memory; BFflex, BRIEF flexibility; BFimp, BRIEF impulsivity; BFbri, BRIEF behavior regulation index; SDQcon, SDQ conduct problems; SDQhyp, SDQ hyperactivity/inattention; SDQext, SDQ externalizing problems; KLCemo, KINDL child emotional well-being; KLPemo, KINDL parent emotional well-being; BFemo, BRIEF emotional control; SDQemo, SDQ emotional symptoms; SDQint, SDQ internalizing problems; KLCfri, KINDL child friends; KLPfri, KINDL parent friends; SDQsoc, SDQ prosocial behavior; KLCtot, KINDL child total well-being; KLPtot, KINDL parent total well-being; SDQtot, SDQ total difficulties.
(TIFF)

S9 Fig Total problems correlations. Numbers indicate Spearman correlations. **p<0.01, ***p<0.001.KLCtot, KINDL child total well-being; KLPtot, KINDL parent total well-being; SDQtot, SDQ total difficulties.
(TIFF)

S10 Fig Externalizing problems correlations. Numbers indicate Spearman correlations. ***p<0.001. BFimp, BRIEF impulsivity; SDQext, SDQ externalizing problems.
(TIFF)

S11 Fig Internalizing problems correlations. Numbers indicate Spearman correlations. *p<0.05, **p<0.01, ***p<0.001. KLCemo, KINDL child emotional well-being; KLPemo, KINDL parent emotional well-being; BFemo, BRIEF emotional control; SDQint, SDQ internalizing problems.
(TIFF)

S12 Fig Social problems correlations. Numbers indicate Spearman correlations. **p<0.01, ***p<0.001. KLCfri, KINDL child friends; KLPfri, KINDL parent friends; SDQsoc, SDQ prosocial behavior.
(TIFF)

## Abbreviations

n-3 LCPUFA: Long-chain n-3 polyunsaturated fatty acids
DHA: docosahexaenoic acid
ADHD: attention-deficit hyperactivity disorder
RCT: randomized clinical trial
EPA: eicosapentaenoic acid
CANTAB: Cambridge Neuropsychological Automated Battery
RTI: Reaction Time
RVP: Rapid Visual Processing
PAL: Paired Associates Learning
SWM: Spatial Working Memory
SDQ: Strength and Difficulties Questionnaire
BRIEF: Behavior Rating Inventory of Executive function
PCA: principal component analysis
CI: confidence interval
OR: odds ratio
