# Supplementary material for: Exploring correlations between neuropsychological measures and domain-specific consistency in associations with n-3 LCPUFA status in 8-9 year-old boys and girls
Source: PLoS One. 2019 May 22;14(5):e0216696. doi: 10.1371/journal.pone.0216696 (PMC6530844; doi:10.1371/journal.pone.0216696)
Supplement: S1 Protocol — (PDF) [file pone.0216696.s001.pdf]

FACULTY OF SCIENCE
UNIVERSITY OF COPENHAGEN

FiSK Junior

The effect of fatty fish and poultry on children's cardiometabolic health and cognitive function

[Effekten af fed fisk og fjerkræ på børns sundhed og kognitive funktion]

STUDY PROTOCOL
H-16018225

Version 3
Date: May 2016

Project Responsible

Associate Professor, PhD Camilla T. Damsgaard
Department of Nutrition, Exercise and Sports
Faculty of Science, University of Copenhagen
Rolighedsvej 26, 1958 Frederiksberg C, Denmark
Phone: +45 3533 2221
E-mail: ctd@life.ku.dk

Clinically Responsible Physician:

Professor, MD, PhD Christian Mølgaard
Department of Nutrition, Exercise and Sports
Faculty of Science, University of Copenhagen
Rolighedsvej 26, 1958 Frederiksberg C, Denmark
Phone: +45 3533 2516
E-mail: cm@life.ku.dk

GCP Coordinator:

Lene Stevner
Department of Nutrition, Exercise and Sports
Faculty of Science, University of Copenhagen
Rolighedsvej 26, 1958 Frederiksberg C, Denmark
Phone: +45 3533 2371
E-mail: less@life.ku.dk

Responsible for cognitive, behavioral and emotional tests:

Associate Professor Lotte Lauritzen, PhD
Department of Nutrition, Exercise and Sports
Faculty of Science, University of Copenhagen
Rolighedsvej 26, 1958 Frederiksberg C, Denmark
Phone +45 35 33 25 08
E-mail: ll@nexs.ku.dk

Assistant Professor Janni Niclasen, PhD (psychologist)
Department of Psychology
University of Copenhagen
Øster Farimagsgade 2A
1353 Copenhagen K
Phone +45 3532 4891
E-mail: janni.niclasen@psy.ku.dk

Funding:

Nordea-fonden
Heerings Gaard
Overgaden neden Vandet 11
1414 København K
kontakt@nordeafonden.dk
Telefon: +45 4060 3040

Place where the study will be conducted:

Department of Nutrition, Exercise and Sports
Faculty of Science, University of Copenhagen
Rolighedsvej 26, 1958 Frederiksberg C, Denmark

TABLE OF CONTENTS

LIST OF ABBREVIATIONS 6

SIGNATURES AND AGREEMENT WITH PROTOCOL 7

1. BACKGROUND 8

1.1 Organization and partners 8
1.2 Scientific background 8
1.2.1 Fish – a nutrient-rich food that we consume too little of 8
1.2.2 n-3 fatty acids and cardiometabolic health 9
1.2.3 Oily fish and cardiometabolic health 9
1.2.4 Cardiometabolic markers in childhood 9
1.2.5 n-3 fatty acids and cognitive function 9
1.2.6 n-3 fatty acids and behavioral and emotional outcomes 10

2. OBJECTIVE 10

3. METHODS 10

3.1 Study design 10
Figure 1. Flow diagram of the study activities 11
3.2 Study foods 11
3.3 Randomization and blinding 12
3.4 Time plan 12
3.5 Study participants 13
3.5.1 Inclusion criteria 13
3.5.2 Exclusion criteria 13
3.5.3 Procedure for exclusion and withdrawal 13
3.6 Recruitment of study participants 14
3.7 Information meeting 14
3.8 Under age study participants 15
3.9 Study activities 15
Table 1. Elements of study activities 16

4. MEASUREMENTS 17

4.1 Anthropometry 17
4.1.1 Weight, height, and waist circumference 17
4.1.2 Body composition 18
4.2 Heart function 18
4.2.1 Blood pressure and heart rate 18
4.2.2 Electrocardiograms of heart rate variability 18
4.3 Cognitive function, behavior, and emotions 18
4.4 Pubertal stage 19
4.5 Dietary intake 19
4.6 Physical activity and sleep 19
4.7 Fasting and standardized breakfast 19
4.8 Biological samples 19
4.8.1 Blood samples 19
4.8.2 Hair sample 20

4.8.3 Salivary sample ... 20
4.8.4 Analysis of biological samples ... 20
Table 2. Biological sample analyses ... 20
4.9 Contact to study participants after completion of the study ... 20

5. STATISTICAL CONSIDERATIONS ... 21
5.1 Statistical power ... 21
5.2 Statistical analysis ... 21

6. COMPLIANCE MEASUREMENT ... 21

7. RISKS, SIDE EFFECTS AND DRAW-BACKS ... 21
7.1 Safety of study foods ... 21
Table 3. Concentration of contaminants in study fish ... 22
7.2 Safety of study procedures ... 23

8. BIOLOGICAL MATERIAL ... 24

9. ETHICAL ASPECTS ... 25
9.1 Compliance with ethical and scientific regulations and guidelines ... 25
9.2 Informed consent ... 25
9.3 Other information provided to the parents ... 26
9.4 Ethical considerations in relation to conducting the study ... 26
9.4.1 Study foods ... 26
9.4.2 Blood sampling ... 26
9.4.3 Fasting ... 27
9.4.4 Puberty questionnaire ... 27
9.4.5 Discomforts from other measurements ... 27
9.4.6 Results from cognitive tests ... 27
9.4.7 Analysis of genetic material (GWAS) ... 28
9.4.8 Serious unexpected adverse reactions or events: ... 28
9.5 Benefits and usefulness of the study ... 28

10. INSURANCE ... 29

11. COMMUNICATION OF RESULTS ... 29

12. MEDICAL ASSISTANCE ... 29

13. REMUNERATION FOR STUDY PARTICIPANTS ... 29

14. FUNDING ... 29

15. REFERENCES ... 31

16. APPENDICES (ALL IN DANISH) ... 37

LIST OF ABBREVIATIONS

Abbreviation	Full name
**ADHD**	Attention-deficit Hyperactivity Disorder
**BDNF**	Brain-Derived Neurotrophic Factor
**CPR**	(Danish) Civil Registration System
**CVD**	Cardiovascular Disease
**DTU FOOD**	National Food Institute, Technical University of Denmark
**EFSA**	European Food Safety Authority
**FiSK**	[Forsknings- og formidlingsprojekt om Fisk, børn, Sundhed og Kognition]<br>Research and communication project on fish, children, health and cognition
**GCP**	Good Clinical Practice
**GWAS**	Genome Wide Association Study
**HbA1c**	Glycosylated haemoglobin
**ID**	Identification
**NEXS**	Department of Nutrition, Exercise and Sports
**PCB**	Polychlorinated Biphenyls
**SD**	Standard deviation
**SNP**	Single Nucleotide Polymorphism
**TEQ**	Toxic Equivalents
**TWI**	Tolerable Weekly Intake
**25(OH)D**	25-hydroxy Vitamin D

SIGNATURES AND AGREEMENT WITH PROTOCOL

The signing academic partners acknowledge that we have read this protocol. We agree to conduct this study in accordance with the study protocol, the current version of the Declaration of Helsinki (64th World Medical Association General Assembly, Fortaleza, Brazil, October 2013), and to strictly enforce all national laws and regulations of the local ethical committee regarding human research.

Christian Mølgaard, M.D, University of Copenhagen, Denmark
Responsible physician of FiSK Junior

Camilla T. Damsgaard, PhD, University of Copenhagen, Denmark
Primary Investigator of FiSK Junior

1. BACKGROUND

1.1 Organization and partners

FiSK Junior, which is described in this protocol, is a research project within the overall research and communication project FiSK [Forsknings- og formidlingsprojekt om Fisk, børn, Sundhed og Kognition] (Research and communication project on fish, children, health and cognition). The overall FiSK project is a partnership collaboration between Department of Nutrition, Exercise and Sports (NEXS), University of Copenhagen and four Danish aquariums: Kattegatcentret in Grenaa, Den Blå Planet in Copenhagen, Fiskeri- og Søfartsmuseet in Esbjerg, and Nordsøen Oceanarium in Hirtshals. The overall aim of the FiSK project is to investigate the potential effects of fatty fish on children's health, cognitive function and behavior as well as communicating our existing evidence-based knowledge on fish intake and human health to Danish families, and to provide playful, fun and inspiring experiences with fish as food. The communication part of project FiSK will be located at the four aquariums, and the research part (FiSK Junior), which is described in this protocol, will be conducted at NEXS.

1.2 Scientific background

1.2.1 Fish – a nutrient-rich food that we consume too little of

The Danish Veterinary and Food Administration recommends that Danes consume 350 g/week fish, hereof 200 g/week fatty fish such as salmon, mackerel and herring (1). Danish 4-9-year-old children have a median intake of about 70 g/week fish and less than 10% of the children reach the recommended weekly intake (2).

Fish is an important dietary source of iodine, selenium, zinc, and high quality proteins, and especially oily fish are rich in n-3 long-chain polyunsaturated fatty acids (n-3 LCPUFA). Oily fish is also an important source of vitamin D, especially during winter when the contribution of vitamin D from sun exposure is negligible in Denmark, and the risk of vitamin D insufficiency is high in both children and adults (3). Intake of n-3 LCPUFA may protect from cardiovascular disease (CVD) in adults (4) and may affect cognitive function and development (5). The potential effects of oily fish on cardiometabolic health and cognitive function have, to our knowledge, not been investigated in children.

1.2.2 n-3 fatty acids and cardiometabolic health

Meta-analyses generally indicate that consumption of fish and n-3 LCPUFA reduce cardiovascular mortality in adults (4,6–8). From randomized controlled trials in adults it is well known that intake of n-3 LCPUFA (typically in the form of fish oil) reduces cardiometabolic risk factors especially blood pressure (9), heart rate (10) and the concentration of triglycerides in plasma (11). Our previous randomized trials with fish oil to infants, toddlers and adolescents indicate that n-3 LCPUFA have comparable effects in children (12–15), but only few studies have investigated this in children.

1.2.3 Oily fish and cardiometabolic health

Randomized controlled trials administering oily fish rather than fish oils have mainly been performed in adults, not in children. These trials show mixed results, but most indicate that consumption of oily fish can improve cardiometabolic risk markers, especially blood pressure and plasma triacylglycerols (16–22). In the OPUS School Meal Study, where more than 700 school children received school meals rich in fish *versus* habitual lunch packs in a randomized design, we previously found that the school meals reduced plasma triacylglycerol, insulin, and blood pressure despite evoking a small increase in waist circumference (23). Further analyses indicated that a main part of the beneficial effects was explained by the increased fish intake (24). However, to our knowledge no randomized trials have investigated the effect of fish consumption *per se* on cardiometabolic health in children.

1.2.4 Cardiometabolic markers in childhood

Although very few children get CVD, elevated cardiometabolic risk markers and metabolic syndrome are seen in childhood (25), and the Bogalusa Heart Study (26) and the Cardiovascular Risk in Young Finns Study (27) demonstrated that blood lipid profile and blood pressure show tracking from childhood and adolescence to adulthood. Therefore, small but persistent reductions in cardiometabolic markers in childhood may be important for long term cardiometabolic risk. As healthy dietary habits, that may evoke such changes, are partly founded in childhood it seems reasonable to promote health and prevent life style diseases through healthy dietary habits from childhood.

1.2.5 n-3 fatty acids and cognitive function

During brain development n-3 LCPUFA are incorporated into the brain, particularly during the first years of life. The rate by which this process occurs has been shown to be affected by diet and is believed to play a role in the cognitive development of the child (28). Most trials investigating the effects of n-3 LCPUFA on cognitive function have been conducted in formula-fed infants or infants of mothers receiving fish oil. Meta-analyses of these randomized trials do not confirm an overall pronounced beneficial effect of n-3 LCPUFA on neurodevelopmental outcomes during the first two years of life (29–31). However, this may be because the effect of n-3 LCPUFA is gender-dependent (32,33). Most studies in young children have used broad developmental scales and have not

examined effects on specific cognitive function, but data indicate that n-3 LCPUFA may specifically affect outcomes related to attention as opposed to memory and processing speed (34,35). Only few studies have examined the effects of n-3 LCPUFA and cognitive outcomes in school children (36–40). However, the studies have mainly been performed in low-income populations, which are quite different from the Danish population, and have mainly used pure n-3 LCPUFA and not fish. Also, the results of these studies have not been analyzed gender-specifically.

1.2.6 n-3 fatty acids and behavioral and emotional outcomes

Dietary n-3 LCPUFA deficiency has been shown to increase motor activity (41) and to affect emotional outcomes such as anxiety and depression, in rodents (41,42). Both pre- and postnatal dietary regimes rich in n-3 LCPUFA have been seen to reduce stress vulnerability and responses to corticosterone (42,43). The described DHA-affected brain functions can be linked to monoaminergic neurotransmitter pathways – *e.g.* anxiety, attention and physical activity, which has been shown to be affected by changes in n-3 LCPUFA intake (41) . A few randomized trials in healthy pre-pubertal school children have showed that children supplemented with fish oil had a more relaxed mood or reduced impulsivity and anti-social behavior compared to control (40,44). Also, lower scores related to traits of attention-deficit hyperactivity disorder (ADHD) have been observed after fish oil supplementation in children living in a low-income area (45). In adults, cross-national data on fish intake indicate that intake of fish and n-3 LCPUFA status play a role in symptoms of depression and patients with depression have been shown to have low n-3 LCPUFA concentrations in their blood and brain (46,47). Therefore, higher habitual dietary n-3 LC-PUFA intake may be protective against mood swings and may prevent mood dysregulation (48). However, as it is not possible to draw any firm conclusions based on the existing studies and further research within this area is needed, especially in children.

2. OBJECTIVE

The main objective of FiSK Junior is to investigate whether intake of fatty fish compared to poultry affects cardiometabolic markers in 8-9 year-old Danish school children. Moreover, we will investigate the potential effects on cognitive function, behavior and emotions.

The study has two primary outcomes: Diastolic blood pressure and plasma triacylglycerol concentration.

3. METHODS

3.1 Study design

The study has a block-randomized controlled 12-week parallel design (**Figure 1**). It will include two groups of 100 children each; one group that receives fatty fish and one group that receives poultry.

The study participants will be 8-9 year-old healthy Danish children who will be enrolled from summer 2016 to April 2017. Apart from an information meeting the study consists of a pre-visit, a baseline visit and an endpoint visit which will be held at Department of Nutrition, Exercise and Sports (NEXS), Rolighedsvej 30, 1958 Frederiksberg C.

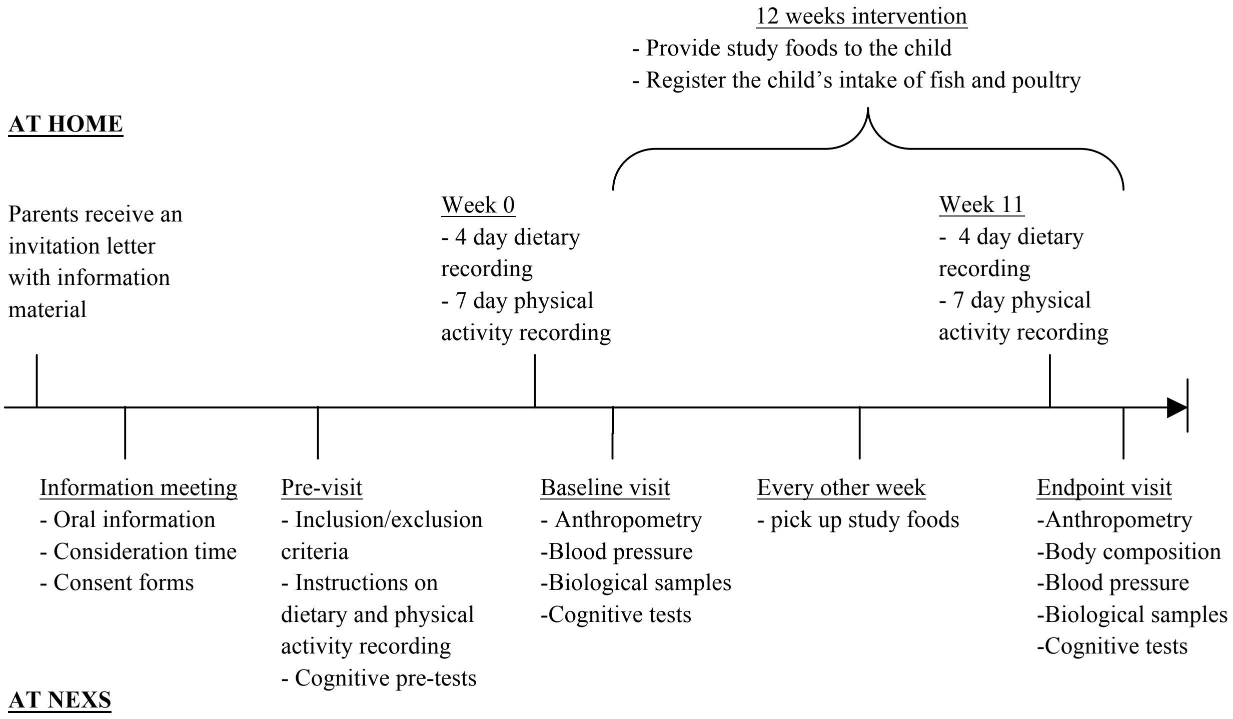

Figure 1. Flow diagram of the study activities

3.2 Study foods

Participants in the intervention group will receive oily fish (with minimum 2 g of fat per 100 g) primarily as fillets of salmon, but also other types of fatty fish such as mackerel in tomato sauce and marinated herring, whereas participants in the poultry group will receive primarily chicken and chicken products (e.g. liver pate, chicken sausage) but possibly also small amounts of duck and turkey. As far as possible, the products provided to the two groups will be matched for energy and fat content. The parents will be instructed that the study fish and poultry should substitute some of the fish and meat usually consumed by the child. Apart from this, the parents will be instructed to maintain the child's usual dietary and physical activity habits during the intervention. The participants will be asked to consume 300 g/week of fish or poultry, operationalized as two times per week as dinner meal and as bread topping / for lunch three times per week, based on typical portion sizes as estimated by the National Food Institute, Technical University of Denmark (49). The goal of 300 g per week is within the national recommendation (1) and will provide

approximately 1 g/day n-3 LCPUFA, which has been shown to reduce blood pressure and plasma triacylglycerol in previous fish oil trials (11,14,15).

Study fish and poultry will be provided as fresh, frozen, and canned foods from NEXS free of charge together with a recipe booklet containing easy recipes and suggestions for preparation of the allocated study foods. The recipe booklet produced for the poultry group will contain poultry recipes resembling those fish recipes provided to the fish group, but with extra oils and fats added when needed to match the fat and energy content of the recipes in the two groups. As most children in this age group eat dinner with their family (50) fish and poultry for dinner will be provided for the whole family/household, whereas lunch products and bread toppings, which are typically eaten in school and more individually, will be provided for the participating child only. The participating child's family will be encouraged to eat the provided study foods for dinner together with the child to enhance compliance. Poultry is chosen as the control food as it is considered to be healthier than the alternative, red meat (51), and has a similar amount of protein and a relatively low content of n-3 LCPUFA compared to oily fish.

3.3 Randomization and blinding

Randomization will be performed in blocks of 12 children, to assure equal allocation to the two study groups over the year. A computer generated randomization list with 200 consecutive 'order numbers' (1, 2, 3,…., 200) will be produced before study start by a staff member not involved in the practical study work. When meeting for the pre-visit each participant will be assigned a unique ID number (in the format D218xxx). At the end the baseline visit the prospective participant is assigned to one of the two intervention groups by the investigator querying the randomization sequence, thus linking participant ID to a number in the randomization sequence and consequently randomly to one of the intervention groups. For example, if the child with ID number D218004 is the second child to finish his baseline visit, he will be assigned to 'order number' 2 on the randomization list, which, according to the list corresponds to e.g. poultry group. This procedure assures optimal data handling and quality, as all test and papers related to the child can be assigned the unique study ID number already from the pre-visit, while at the same time assuring that group allocation is not performed until after the baseline measurements. This procedure ensures blinding of both participants and investigators until the baseline measurements have been completed. Due to the taste and nature of the intervention foods, blinding of study staff and participants is not possible through the entire study.

3.4 Time plan

Participants will be enrolled from summer 2016 to April 2017. The baseline visits will be conducted during August 2016 - April 2017. Endpoint visits will be conducted during November 2016 - June 2017. The duration of the intervention period will be 12 ± 3 weeks.

3.5 Study participants

3.5.1 Inclusion criteria

Children must:

- Be 8 - 9 years of age at the start of the intervention
- Be healthy
- Like oily fish and chicken
- Be willing to be randomized to eat fish or chicken
- Not consume fatty fish more than once per week 3 months prior to intervention start
- Not consume fish oil 3 months prior to intervention start
- Speak Danish in order to understand the study procedures

Moreover:

- Parents must read and speak Danish, in order to be properly informed about the study procedures
- The child must live in a household with ≤5 persons

3.5.2 Exclusion criteria

Exclusion criteria for the children:

- Serious chronic illnesses and diseases that may interfere with study outcomes
- Diagnosed ADHD or other psychiatric illness
- Intake of medication that may affect study outcomes
- Concomitant participation in other studies involving dietary supplements or blood sampling
- Living in a household with another participating child

3.5.3 Procedure for exclusion and withdrawal

A participant can be excluded from the study if the clinically responsible physician finds it advisable due to medical reasons. A participant can also be excluded for ethical reasons e.g. if the project staff and/or clinically responsible physician judges that the child does not want to continue his/her participation in the study, which will always be respected. Finally, a participant can be excluded if non-compliance with the procedures described in the information material is clearly demonstrated. Reasons for exclusion will be recorded and parents will be informed about the decision.

Parents will be informed that they can withdraw their child from participation at any time without giving a reason. At withdrawal we will ask the parents if they will tell us the reason for withdrawal. Responding is voluntary and we will not put pressure on the parents to make them respond.

3.6 Recruitment of study participants

A total of 150 children completing the study are needed. From our previous studies in children we expect a drop-out of up to 15-20%, and expect unsuccessful blood sampling in an additional 10% of the children at either baseline or endpoint. Therefore, a total of 200 participants will be recruited.

Boys and girls of the relevant age will be identified through the Danish Civil Registration System (CPR registry) and recruited via postal invitation letter to the parents (Appendix 2). Participants will mainly be recruited from the Capital Region of Denmark, but if necessary also from other parts of Zealand. Participants may also be recruited through advertisements in Danish newspapers, shared through networks and social media such as Facebook, and by notices in schools and after school clubs (Appendix 6). Parents that respond to the advertisements will receive the same information material as parents contacted through the CPR registry. The letter will contain a link to an electronic response form and the information material (Appendix 3). The brochure ”The legal rights of study participants in a health science research project” [Forsøgspersonens rettigheder i et sundhedsvidenskabeligt forskningsprojekt], published by the Danish Committee on Biomedical Research Ethics system in August 2014, will be included in the information material.

Parents are asked to carefully read the material, if they wish together with a family member or a friend, and to explain the content to their child. Parents who return the response form will be called by telephone by the project staff. Here the participants will be pre-screened according to the inclusion and exclusion criteria. If they are still interested in participating, they will be invited to an information meeting where the study will be explained orally to parents and child by a project staff member who has been specifically trained for this task. Parents will be informed that they can bring an assessor (e.g. a family member or a friend) to the information meeting, if they wish so. If the participants do not fulfil the criteria for participation they will be informed hereof and will not be contacted or bothered further. Only one eligible child from each household can be included as a study participant, since for practical reasons individual randomization within a household is not possible.

3.7 Information meeting

Information meetings will be held individually or in groups of max. 10 families in a dedicated room by one or more project staff members. At this meeting the parts of the study that involves the family and child will be explained in detail and there will be time and room for asking questions. Parents will be informed that they and the potential assessor can ask questions to a project staff member, who has been delegated for this task in a separate room one to one. If the project staff judges that some parents need a second explanation of the study, this will be encouraged. It will be emphasized that the participants have the right to consideration time of at least 24 hours, that participation is voluntary, and that the participants can withdraw from the study at any time without giving a reason.

In case they want to participate and do not wish further consideration time, both parents/custody holders can fill out the consent form for the child and for being invited for potential future follow-ups of the study (Appendix 4a) and for the biobank (Appendix 4b), if they wish to allow storage of possible excess material of blood in the biobank. Consent to donate biological material for the biobank and consent to allow us to contact the families in relation to future follow-ups of the study are not required for participation. The project staff will explain the content of the consent forms to the parents. If the parents give written consent to be invited for potential future follow-up of the study, they will only be invited after the potential follow-up study has been approved through a supplementary protocol submission to the Danish Regional Committee on Biomedical Research Ethics.

When both parents or all custody holders of the child as well as the project responsible (or a project staff member who have had this responsibility assigned by the project responsible) have signed the consent form(s), parents are offered a copy of the signed form(s). One parent or custody holder can empower by signature the other parent/ custody holder to decide whether the child should participate (Appendix 5). There will not be conducted any project activities before written informed consent has been given.

3.8 Under age study participants

The study participants are under age. We consider the study to be substantiated according to Committee Regulations [Komitelovens § 19, stk. 3] regarding trials not involving drugs, since the study can only be conducted in the described age group in order to answer the relevant research questions. Also, the study confers minimal risk of harm for the participants.

According to Committee Regulations [Komitelovens § 5, stk. 2] the information to the study participant needs to be suited for the apprehension of the participant, and information to under age participants has to be given by a person with understanding of the area of study and relevant pedagogical skills for the age group. At the information meeting the study and study activities will be explained to the child in a language and at a level that is appropriate and suited for the age and apprehension of the child, and with use of visual images if required, by a project staff member with experience in communicating to children.

As the study participants are under age they cannot give informed consent. However, the project staff informing and talking with the child will be aware of the child's response. If the project staff judges that the child does not want to participate in the study, the child will not be included.

3.9 Study activities

The different elements of the study are listed in **Table 1**. During the study we will send the parents reminders by text message and/or email; this procedure will be described at the information

meeting. We will make sure to plan the 4-5 hour visits at NEXS with appropriate breaks for the child including time for breakfast that will be provided to the child.

Table 1. Elements of study activities

**Information meeting at NEXS (1 hour)**	• An understandable presentation of the context and purpose of the study<br>• Explanation of the study procedures and measurements.<br>• Any foreseeable risks, side effects, complications and inconveniences<br>• Unpredictable risks<br>• The right for time to reflection<br>• Volunteering<br>• Confidentiality<br>• The right to decline information on the study results<br>• That all data collected during the study will be used in the event of a participant chooses to withdraw from the study<br>• Description of the study products/food<br>• Review of the pamphlet "Before you decide" published by DNVK August 2014<br>• Questions from the parents<br>• Explanation of informed consent and withdrawal
**Pre-visit at NEXS (1 hour)**	• Informed consent<br>• Inclusion/exclusion criteria<br>• Instructions on 4-day dietary recording<br>• Instructions on accelerometer measurements<br>• Cognitive pre-testing
**At home before baseline visit**	• Fill out lifestyle questionnaire (Appendix 7)<br>• Fill out questionnaire on puberty<br>• Fill out questionnaire(s) on behavior, emotions, and cognitive function (Appendix 9-11)<br>• 4 days of dietary recording<br>• 7 days + 8 nights of physical activity and sleep recording (accelerometer)<br>• Apply EMLA patches to the child's forearms<br>• Overnight fasting
**Baseline visit (4-5 hours)**	• Empty bladder if possible
Cardiovascular function	• Blood pressure and heart rate<br>• Continuously measured heart rate by electrocardiograms
Anthropometric measurements	• Height<br>• Weight<br>• Waist circumference<br>• Body composition
Biological samples	• Hair strands

	• Blood sample<br>• Salivary samples
Other	• Child has breakfast
Cognitive function, behavior and emotions	• Tests, interviews and questionnaires
Study foods	• Interview on dietary habits, preferences, and perceptions<br>• Instructions on intake and recording of study foods<br>• Receive first batch of study foods
**During the intervention (12 weeks)**	• Collect study foods from NEXS (every 1-2 weeks)<br>• Cook and eat study foods<br>• Register intake of study foods
**At home before endpoint visit**	• Fill out lifestyle questionnaire (Appendix 8)<br>• Fill out questionnaire on behavior, emotions, and cognitive function (Appendix 9-11)<br>• 4 days of dietary recording<br>• 7 days + 8 nights of physical activity and sleep recording (accelerometer)<br>• Apply EMLA patches to the child's forearms<br>• Overnight fasting
**Endpoint visit (4-5 hours)**	• Empty bladder if possible
Cardiovascular function	• Blood pressure and heart rate<br>• Continuously measured heart rate by electrocardiograms
Anthropometric measurements	• Height<br>• Weight<br>• Waist circumference<br>• Body composition
Biological samples	• Hair strands<br>• Blood sample<br>• Salivary samples
Other	• Breakfast
Cognitive function, behavior and emotions	• Tests, interviews and questionnaires
Study foods	• Interview on dietary habits, preferences, and perceptions

4. MEASUREMENTS

4.1 Anthropometry

4.1.1 Weight, height, and waist circumference

The child will be weighed on a digital scale with the child in underwear. Standing height will be measured three times to the nearest millimeter with a stadiometer. Waist circumference will be measured three times to the nearest millimeter by a non-elastic measuring tape at the level of the umbilicus. The mean of three measurements will be used for height and waist circumference.

4.1.2 Body composition

Fat mass and fat-free mass will be measured by bio impedance with the child lying down without moving after 10 minutes rest, if possibly with empty bladder. Four electrodes will be placed at the dorsal surface of the right hand and foot. The measurement takes about 2 minutes after electrodes have been placed. Fat mass will also be measured by three repeated skinfold measurements at triceps and subscapularis, respectively, while the child is standing.

4.2 Cardiovascular function

4.2.1 Blood pressure and heart rate

Blood pressure and heart rate will be measured three times by an automated device with the child lying down after 10 minutes rest. The appropriate cuff size for the child's arm circumference will be used. This procedure will be repeated up to four times during the five hour baseline and endpoint visits to account for potential variation in blood pressure during the visits and thereby obtain the most accurate measure of the primary outcome.

4.2.2 Continuously measured heart rate by electrocardiograms (ECG)

Throughout the baseline and endpoint visits, electrocardiograms will be recorded continuously by a Holter monitoring recorder device. When the child has arrived at the department four electrodes will be placed on the chest of the child. Electrodes will be removed again by the end of that examination visit. The recordings will be analyzed at Department og Clinical Medicine, Aalborg University Hospital, by Jeppe Hagstrup Christensen (Clinical Professor, MD).

4.3 Cognitive function, behavior, and emotions

We will use a number of computer (e.g. Flanker's and test from the CANTAB battery: http://www.cambridgecognition.com/academic/cantabsuite/tests) and paper based test (e.g. Stroop, and D2 (Appendix 12)) to assess specific areas of cognitive function focusing on memory, attention, and reaction time. The tests are internationally recognized, validated and standardized tests that will be adjusted to the children's age. Children's physiological stress response will be assessed using a cold pressor test, where the child submerges his/her dominant hand in a tub containing water (temperature 5°C±2°C) for maximum 1 minute. Before and immediately after the test we measure the child's blood pressure and heart rate, and before and 20 min after the test we collect a salivary sample to be analyzed for cortisol concentration. Together with the heart rhythm recorded continuously during the test (see 4.2.2), these outcomes will be used to assess the child's overall physiological stress response. Children are instructed that they can remove their hand from the water if they find the test too uncomfortable and will be reminded about this during the test if they display discomfort. Cognitive, behavioral and emotional outcomes will also be measured by validated questionnaires to be filled in by the parents (Appendix 9-11). A pre-test prior to the baseline visit will be performed for some of the cognitive tests to avoid interference by learning effects. Prior to testing the project staff will instruct the children on how to do the tests.

Trained project personnel will conduct interviews with the children based on the Kid-KINDL questionnaire (Appendix 20) assessing children's quality of life. Parents might be asked to fill in some of the questionnaires at home prior to the baseline and endpoints visits to assure that the same

parent answer the questions at both visits (Appendix 9-11). Assessments of cognitive function, behavior, and emotions will be supervised by psychologist, PhD, Janni Niclasen.

4.4 Pubertal stage

At baseline pubertal stage will be evaluated by the parents, if necessary aided by the child, using a self-administered questionnaire on breast development and age at menarche for girls (Appendix 13) and pubic hair for boys (Appendix 14).

4.5 Dietary intake

The children's dietary intake will be recorded by the parents prior to the baseline and endpoint visits during four days of weighed dietary recording registered in a web based program (www.madlog.dk). Parents will be instructed on how to weigh and record the children's dietary intake during the pre-visit at NEXS (Appendix 15). If weighing of the food is not possible, parents will be asked to indicate household measures. If the parents do not have a digital kitchen scale at home it will be possible to borrow one from the study site. Also, during the 12 week intervention period the parents will be asked to register the children's intake of study products weekly. The children's habitual intake of fish, poultry and meat will be examined by a food frequency questionnaire (Appendix 19) filled in by the parents before the baseline and endpoint visits.

4.6 Physical activity and sleep

The children's physical activity and sleep will be measured by accelerometers during 7 consecutive days and 8 nights prior to the baseline and endpoint visits. During the pre-visit parents and children will be instructed on how to use the accelerometers (Appendix 17).

4.7 Fasting and standardized breakfast

The children will be asked to meet for the baseline and endpoint visits fasting (Appendix 18). That is they are not supposed to consume foods or drinks with calories from midnight the night before the visits. After the cardiometabolic measurements breakfast will be provided to the child. The same type of breakfast will be provided at the baseline and endpoint visit to ensure minimum influence on the subsequent cognitive measurements. If a child does not like the breakfast served he or she is allowed to bring breakfast from home, but the family will be instructed to bring the same type of breakfast at both visits.

4.8 Biological samples

4.8.1 Blood samples

Before the baseline and endpoint visits parents will receive local anesthetic EMLA patches with instructions on how to apply them (Appendix 18). At each visit 40 ml venous blood will be drawn from the child's forearm after removal of the patches. Samples will be processed and stored at NEXS at –80°C until analysis. Following analysis the remaining samples will be destroyed, unless separate consent for storage of remaining blood in the biobank has been given.

4.8.2 Hair sample

A small tuft of hair (approximately 5-20 mg) will be cut with a pair of scissors from the posterior vertex of the child's head. The three centimeters of the hair strands located most closely to the scalp will be used for analysis of cortisol, reflecting cortisol levels over the last 3 months.

4.8.3 Salivary sample

The salivary samples (2 x approximately 0.5-2 ml) will be collected before and after the cold pressor test using a small swab which the child will chew for about 60 seconds to stimulate salivation. Saliva samples will be centrifuged and stored at NEXS at maximum –20°C until analysis of cortisol. The salivary cortisol level reflects the acute serum cortisol level.

4.8.4 Analysis of biological samples

Table 2 gives an overview of the planned analyses of the biological samples.

Table 2. Biological sample analyses

Samples	Field	Analyses
**Blood samples**	**Cardiometabolic**	• Triacylglycerol and cholesterols<br>• Glucose, insulin, and glycosylated haemoglobin (HbA1c)<br>• C-reactive protein, adipokines, and related markers
	**Compliance**	• Fatty acid composition and lipid species
	**Nutrient status**	• Serum 25(OH) vitamin D, vitamin D metabolites and binding proteins<br>• Haemoglobin and ferritin<br>• Other nutrient biomarkers
	**Immune**	• Immune cells, cytokines and other immune markers
	**Hormones**	• Sex hormones, appetite hormones
	**Growth**	• Bone metabolites, growth factors and hormones
	**Neurocognition**	• Blood markers related to brain function such as brain-derived neurotrophic factor (BDNF) and serotonin
	**Genetics**	• Epigenetics and genes related to the study outcomes, and research questions (single nucleotides polymorphisms (SNPs) and genome wide association studies (GWAS), NOT full genome sequencing)
**Hair strands**	**Stress**	• Cortisol and related metabolites
**Salivary samples**	**Stress**	• Cortisol and related metabolites

4.9 Contact to study participants after completion of the study

After the participant has completed the 12 weeks intervention period the study staff members may contact some of the parents to the participating children to ask whether the child/parents would like to participate in small videos and interviews about the research project. Participation in these activities is completely voluntary and has no influence on the participation in the research project.

5. STATISTICAL CONSIDERATIONS

5.1 Statistical power

The primary outcomes of the study will be diastolic blood pressure and plasma triacylglycerol. Power calculations were performed using data from our previously conducted OPUS School Meal Study (23). With 150 completing children we will have 80% power to detect a difference of less than 0.5 SD of the outcomes i.e. 2.7 mmHg in diastolic blood pressure and of 0.13 mmol/L in triacylglycerol between the two intervention groups, respectively. This would correspond to approximately 4% reduction in blood pressure and 20% reduction in plasma triacylglycerol among this age group, which is considered relevant and obtainable.

The cognitive measurements will be secondary and exploratory outcomes, and therefore not subject to power calculation. However, in the OPUS School Meal Study we previously found that school meals rich in fish improved school performance and reading comprehension in Danish 8-11 year-olds (52). Although the sample size was about 4 times larger in that study, the fish group of FiSK Junior will get 5-10 times more n-3 LCPUFA than in the school meal study, and therefore we expect FiSK Junior to have a sufficient sample size for the cognitive outcomes as well.

5.2 Statistical analysis

The effect of the intervention on the outcomes will be evaluated using analysis of covariance including baseline outcome values as covariate. Outcomes will also be analyzed with gender and genotype as potential effect modifiers.

6. COMPLIANCE MEASUREMENT

The content of n-3 LCPUFA in erythrocytes will be used as biomarker of compliance, reflecting intakes over the last 3-4 months. Also, during the 12 weeks intervention period parents will be asked to register the child's intake of study foods as another measure of compliance.

7. RISKS, SIDE EFFECTS AND DRAW-BACKS

There is no substantial risk associated with participation in the intervention.

7.1 Safety of study foods

Apart from healthy nutrients, fish may contain unwanted substances stemming from human and natural contamination of the seas. Therefore we have thoroughly considered the safety aspects of

the study foods in collaboration with senior advisor and toxicologist Max Hansen, Division of Risk Assessment and Nutrition, National Food Institute, Technical University of Denmark (DTU FOOD). Two groups of substances are of special interest in relation to safety of fish intake: 1) Mercury and 2) Dioxins and dioxin-like polychlorinated biphenyls (PCB). Children in the intervention group will consume up to 300 g oily fish per week and the main types provided will be aqua cultured salmon (the most commonly eaten source of salmon in Denmark), some mackerel and small amounts of herring. All will be products that are available in Danish supermarkets and commonly eaten by Danes. The content of mercury and toxic equivalents (TEQ) of dioxin and dioxin-like PCB in these fish types are shown in Table 3. Mercury is mainly an issue in large predator fish such as tuna, shark and ray fish, which will not be used in the study.

Table 3. Concentration of contaminants in study fish

	**Mercury** (mg per kg fish)	**Dioxin and dioxin-like PCB** (ng TEQ per kg fish)
Aqua cultured salmon	0.021 (0 - 0.059)	0.57 (0.35 - 1.4)
Mackerel	0.039 (0.009 - 0.36)	0.87 (0.12 - 9.7)
Herring	0.05 (0.010-0.23)	1.2 (0.27-5.2)

Data is shown as upper bound mean (min-max). Upper bound mean implies that concentrations below the limit of detection have been substituted with the value of the limit, i.e. a likely overestimate. Values for salmon are from (53); values for mackerel and herring are from (54).

Assuming an intake of 200 g salmon + 80 g mackerel + 20 g herring every week the children will have an intake of 8.3 µg mercury and 208 pg dioxin and dioxin-like PCB per week. The European Food Safety Authority (EFSA) has established a tolerable weekly intake (TWI) for these substances, i.e. the estimated amount that can be ingested weekly over a lifetime without appreciable health risk. The TWI is 1.3 µg/kg bodyweight for methyl-mercury (55) which is the most common form of mercury in marine foods. The TWI for dioxin and dioxin-like PCB is 14 pg TEQ/kg bodyweight (56). Danish children 8 years of age have a median bodyweight of 27.5 kg (57,58). Assuming that all mercury in the fish is methyl-mercury, the TWI for the youngest children in the study will therefore be 1.3 µg/kg × 27.5 kg = 36 µg per week for methyl-mercury and 14 pg TEQ/kg × 27.5 kg = 385 pg TEQ per week for dioxin and dioxin-like PCB. Hence the study fish will provide 23% and 54% of the children's TWI for mercury and for dioxin and dioxin-like PCB, respectively.

Fish is the main source of mercury in the general Danish diet, whereas fish contributes about 30% of the intake of dioxins and PCB in the general population (59). The remaining 70% comes from milk, cheese, meat, and fats. DTU-FOOD has estimated the total mean intake of dioxin and PCB among Danish 4-14 year-olds to be 0.87 pg/day per kg bodyweight. As fish contributes 30% to this intake the children in the study are expected to have an intake of 0.61 pg/day per kg bodyweight or 4.3 pg/week per kg body weight from non-fish sources. The contribution from the study fish will be an estimated 208 pg/week per child / 27.5 kg = 7.6 pg/week per kg body weight. We assume that the study fish will be the only source of fish in the children's diet. The total intake of dioxin and dioxin-like PCB will therefore be 4.3 + 7.6 = 11.9 pg/week per kg bodyweight which is well below

the TWI of 14 pg/kg bodyweight. In agreement with the risk analyses conducted by DTU-FOOD in this field, we expect that the variation in the contribution to the intake of dioxin and dioxin like PCBs from the study fish will be small, i.e. for almost all children the actual intake will be close to 208 pg/week. Moreover, the families will be instructed that the study fish must replace some of the meat in the diet, which in Denmark contribute with 18% to the total intake of dioxin and dioxin like PCB. Therefore the actual intake of dioxin and dioxin-like PCB is likely to be even lower than calculated. **Altogether, it is unlikely that children in the fish group will have an intake of mercury or dioxin and dioxin-like PCB that exceed the tolerable weekly intake (TWI) during the intervention.**

Children in the control group will consume up to 300 g poultry as part of the study, mainly chicken as chicken legs and breast, but also other products such as chicken liver pâté and sausage, i.e. products that are available in most supermarkets and commonly eaten by Danes. Poultry do not contain significant amounts of harmful substances. Danish 4-9 year-olds consume on average almost 600 g (5) meat and meat products per week (2). As in the fatty fish group, families in the control group will be instructed to substitute the poultry and poultry products for some of the meat and fish normally eaten, to avoid an overly high protein intake.

In conclusion, the study foods are therefore considered safe for the participants.

7.2 Safety of study procedures

Blood samples will be taken by experienced laboratory technicians who are used to taking blood samples in children, and the only expected risk in relation to blood sampling is the risk of a mild pain and potentially a small bruise at the site of the prick, which will disappear within a few days. The volume of the blood sample is small (40 ml per sample) and the two blood samples are taken three months apart. To reduce the discomfort of blood sampling we will provide local anesthetic patches (EMLA) to the parents, containing lidocaine and prilocain, to put on both forearms at home before the blood sampling. The patches are sold over-the-counter in Danish pharmacies.

Bio impedance is non-invasive and has not been reported to give untowards events. A very small electric current is sent through the body, however it is lower than the threshold of perception and unlikely to stimulate electrically excitable tissues such as heart and cardiac muscle. The method has been approved by the US Food and Drug Administration.

The cold pressor test generally causes some cold-related pain, but this risk is balanced by the participants' control over the process (i.e., their ability to withdraw their hand if it becomes too uncomfortable) and the fact that the cold-related pain mounts fairly slowly so that it can be terminated before it becomes too uncomfortable (60). As a further means of minimizing risk, the following guidelines published by Von Baeyer and colleagues (2005) will be followed: Children with a history of cardiovascular disorder, fainting or seizures, frostbite, open cut, sore or fracture of hand to be immersed, and any history of Reynaud's phenomenon (hands get white, then blue, on

exposure to cold, then red on warming), will be excluded from the cold pressor test. In addition, the children will have breakfast before the test to reduce risk of a vasovagal stress response (fainting) (61). A review of cold pressor test studies with an estimated total of 3000 child participants only reported two adverse events involving significant but transient distress (60). In our study of 200 children we therefore consider the risk of an adverse event minimal. However, we will pay close attention to the reaction of the child, and if a child experiences severe distress or displays other symptoms, we will terminate the test, comfort the child and immediately call their parent.

The study foods and activities are not expected to cause other side-effects or harms. As in any other research projects there is a theoretical risk of unexpected events or risks, which cannot be foreseen by investigators or study participants and therefore this study cannot exclude risks which are unknown at this time. If we suspect or the participants report any side effects, harms or diseases during the study, the medically responsible physician will determine whether the study participant can continue in the study and will inform the parents/ custody holders. If we obtain new information about the procedures or intervention products that are of major relevance to the participants, the parents/ custody holders will receive written and oral information hereof and will have to give renewed written consent if they want to continue participation in the study.

8. BIOLOGICAL MATERIAL

In connection with the study, a research biobank will be established for storage of biological material, that is blood (2 x 40 ml), hair samples (2 x 5-20 mg), and salivary samples (4 x 0.5-2 ml) for each child. The whole project and the research biobank are reported to the Danish Data Protection Agency via common reporting system in the Faculty of SCIENCE [fællesanmeldelse], ref. no. 2015-57-0117. Biological material stored in the research biobank will be destructed after analysis and no later than 7 years after study completion. Samples will be analyzed at NEXS and at other laboratories in Denmark, other EU countries, USA, and Canada. After shipping to these countries samples will be treated in agreement with the national laws of the recipient country. Samples will be sent in coded tubes without any accompanying data files that could potentially identify the participants.

Possible excess material of blood will, if the parents/custody holders have agreed to this, be transferred to a biobank; otherwise it will be destroyed. The biobank material will be stored at -80˚C at Department of Nutrition, Exercise and Sports, Rolighedsvej 26, 1958 Frederiksberg C, Denmark, for max. 7 years after study completion. The material will only be used in new and related research projects after these have achieved approval by the Danish Regional Committee on Biomedical Research Ethics, unless the samples have been totally anonymised, this means that the ID-log that connects study participants and biological material is destroyed. If the parents/custody holders choose to donate possible excess material to the biobank and later on regret it, they can contact Department of Nutrition, Exercise and Sports and have their child's blood destroyed. Parents/custody holders will, according to the procedures at Department of Nutrition, Exercise and Sports, sign a separate consent form for the biobank. Parents/ custody holders will be informed that

it is completely optional if they want to donate excess material from the child to the biobank and that the decision has no consequences for the participation in the study. The applicant is aware of the fact that collection of biological material for a biobank is not covered by the committee law, but by the Danish Act on Processing of Personal Data *(Persondataloven*). As we consider the procedure relevant for the complete research protocol in Denmark, the information is included in the material for the committee.

9. ETHICAL ASPECTS

9.1 Compliance with ethical and scientific regulations and guidelines

- The study and recruitment will not be initiated until approval has been obtained from the Danish Regional Committee Biomedical Research Ethics. The written approval and potential appendices will be kept at NEXS, where the study will be conducted. If any major changes are made to the protocol or the essential appendices to the protocol, these will be submitted to the Committee as a supplementary protocol.
- We will follow the Danish Act on Processing of Personal Data [Persondataloven] for all data collected in the study. The study as a whole is reported via the common reporting system in the Faculty of SCIENCE [fællesanmeldelse], ref. no. 2015-57-0117.
- The study will be conducted in agreement with the ethical principles described in the latest, updated version of the Helsinki Declaration (62) and will be registered in the database www.clinicaltrials.gov before recruitment is initiated.

9.2 Informed consent

The parents or custody holders of the child will be informed orally and in writing about the study, and have to be legally able to give written consent for the child's participation. Parents/ custody holders can withdraw their consent for participation at any time without giving explanations and without any consequences for them or the child. Parents will be informed that data, that was collected before withdrawal, may be used by the investigators in the data analysis of the study. If they do not wish this use of their data, they must tell us to delete their data from the data set. The informed consent gives all relevant authorities that regulate the study, such as the Danish National Committee on Biomedical Research Ethics, consent to see the data of the participants.

We will not perform any study activities until both parents or all custody holders have given written consent based on oral and written information on:

- The study and its activities and procedures
- Their right to withdraw from the study at any time without giving explanations and without consequences
- The procedures for collection and storage of the personal and biomedical data
- That the personal and biomedical data will be processed anonymously

9.3 Other information provided to the parents

During and after the study the parents will be informed if:

- The study responsible becomes aware of any new information on effects, risks or harms related to the intervention
- The design of the study is changed substantially
- The study team members discovers any important health information about their child (unless they have specifically declined to receive such knowledge)
- The study is discontinued; in this they will be informed about the reasons for the discontinuation

If the project staff suspects that a child is ill during the information meeting or during the study or suspect any side-effects of participating in the study, the medically responsible physician will determine whether the participant can continue in the study and inform the parents/ custody holders. If the parents wish so, they will be informed about the results of these investigations.

9.4 Ethical considerations in relation to conducting the study

9.4.1 Study foods

The study foods that will be used are fish and poultry such as chicken cuts, filets and products that are commercially available in Danish supermarkets and commonly eaten by Danes. Intake of 300 g/week of fatty fish is within the national dietary recommendation on fish intake (1). As described in section 7.1 we have thoroughly considered the safety aspects of children's consumption of the study foods for 12 weeks, particularly in relation to potential contaminants in the fish. Based on these calculations and the tolerable intakes of unwanted substances defined by EFSA, we conclude that the study foods are safe for the participants.

9.4.2 Blood sampling

We have thoroughly considered the ethical aspects of the discomfort of blood sampling. Since the blood samples are crucial for investigating one of the primary outcomes (triacylglycerol) and compliance (n-3 LCPUFA) and since this investigation is of major importance for qualifying or improving dietary fish recommendations for children and investigating potential cognitive, behavioral and emotional effects of fish, we consider the discomfort of the blood sample to be outweighed by its usefulness. The discomfort of blood sampling will be minimized by use of local anesthetic patches on the forearms before blood sampling. Moreover, as described in section 7.2, blood will be sampled by laboratory technicians who are experienced in blood sampling in children. The only expected risk of blood sampling is a mild pain at the site of the prick on the arm and potentially a small blue bruise at the site of the prick, which will disappear within a few days.

9.4.3 Fasting

The children will be asked to meet at the baseline and endpoint visit fasting. The cardiometabolic examinations are conducted in the morning, and the children will be served a breakfast afterwards before the cognitive tests. A fasting period of this duration is relative short, and we do not expect the children to experience considerable discomfort due to the fasting. However, if this should be the case, the examinations will be discontinued immediately and the child offered drinks and food.

9.4.4 Puberty questionnaire

As described in section 4.4 the parents will be asked to report the child's pubertal stage, aided by the child if necessary. The recording is scientifically important as pubertal onset affects body composition and a number of the blood values to be measured. The parent(s) will fill out the questionnaire at home or at the baseline visit. A member of the study team will be available for answering questions, but will not otherwise partake in this activity unless asked to, in order to assure participant privacy.

9.4.5 Discomforts from other measurements

Some children may be slightly irritated by wearing the accelerometer, but the children can take off the accelerometer if annoyed too much. Some children may also be a bit nervous about having a small tuft of hair cut off. However, the amount of hair needed to measure cortisol is very small and the project staff will assure that the hair strands is cut from the back of the child's head in the least visible way. Some children may experience a mild discomfort from a "pinch" while having skinfolds measured and from lying down without moving during the bio impedance measurement. The study team members will do these measurements as quickly as possible to minimize the potential discomfort. The children will generally experience some discomfort during the cold pressor test due to the cold-related pain, but they are able to remove their hand at any time, and the pain mounts slowly and can be terminated before becoming severe. We will pay close attention to the reaction of the child and if the child experiences distress or displays other symptoms, we will terminate the procedure, comfort the child and call their parent.

9.4.6 Results from cognitive tests

The purpose of the assessments of cognitive function, behavior, and emotion is to describe variations within certain areas of cognition and behavior in healthy children in the two intervention groups. The tests will not be used to diagnose or make a general evaluation of the individual child. The personnel performing the measurements will be trained and supervised by a psychologist to assure the most comfortable and proper test environment for the children.

9.4.7 Analysis of genetic material (GWAS)

The blood stored in the research biobank will also be used for genome wide association studies (GWAS) analysis, analysis of single nucleotide polymorphisms and epigenetics. It is highly unlikely that the analyses will detect any serious inheritable genetic disposed illnesses. The genotype analyses will not include any full genome or exome sequencing, but will use a chip, that reveals information on common gene variants (>5%). It is therefore considered highly unlikely that we identify any genetic variants that have any direct relevance for illnesses.

9.4.8 Serious unexpected adverse reactions or events:

A serious adverse event is any untoward medical occurrence that:

- Results in death
- Is life-threatening at the time of the event;
- Requires inpatient hospitalization;
- Results in persistent or significant disability or incapacity.

The project responsible will immediately inform the committee if suspected unexpected serious adverse reactions or serious events occur during the project. The report will comment on any outcomes for the concerned trial. Reporting will take place no later than 7 days after the project responsible became aware of any such adverse reactions or events, as well as once yearly during the study together with an assessment of the safety of the study participant. The standard forms from the committee will be used for this purpose. In case of serious adverse reactions or serious events resulting from the project, the investigator will make available any information requested by the committee.

At the baseline visit parents will be made aware to contact us throughout the study period in case they experience any adverse events or reactions, and at the endpoint visit parents will be asked if they have experienced any adverse events or reactions. All adverse events and reactions will be registered and evaluated using a standard form from the Danish GCP unit (Appendix 21).

9.5 Benefits and usefulness of the study

The participating children and parents will get insight into and contribute to research in child nutrition and health with potential impact on dietary recommendations for children. After completion of the study the parents will receive their child's vitamin D status. Also, after completion of the study, data analysis, and writing of main scientific papers the parents will receive an the overall main conclusions from the study.

The study will fill in the knowledge gaps on the effect of fish intake on cardiometabolic health in healthy Danish children. Moreover, the study can elucidate potential effects of fish on cognitive function, behavior, and emotions which are also important for our understanding of the role of fish

and n-3 fatty acids in child health. The results can contribute to qualifying or revising the dietary recommendations for children in relation to fish intake as these are primarily based on studies conducted in adults. Collectively, the study is judged to be ethically justified and proper as the individual, scientific, and societal benefits are believed to outweigh the limited discomfort and very low risk of harm associated with the study.

10. INSURANCE

The study participants are covered by the legal act [*Lov om arbejdsskadeforsikring*] in referral to the existing insurances at Faculty of Science, University of Copenhagen. During the study the participants are covered by the legal act [*Lovbekendtgørelse 1113 ad 7 nov 2013*] (www.retsinformation.dk).

11. COMMUNICATION OF RESULTS

The study will be registered in the publicly accessible trial database www.clinicaltrials.gov. Authorships will be decided on in agreement with the Vancouver Declaration. Both positive and negative as well as inconclusive findings will be published. Articles will be sought published in international scientific peer-reviewed journals and communicated through other relevant media.

12. MEDICAL ASSISTANCE

Professor, MD Christian Mølgaard can be contacted and will provide medical assistance if needed during the study. Moreover, in the examination area there is a list of names and phone numbers of medical doctors employed at NEXS and working at Frederiksberg Campus who can be contacted and provide medical assistance and guidance if needed.

13. REMUNERATION FOR STUDY PARTICIPANTS

At the endpoint visit the participating child will receive a free annual pass [årskort] to be used in one of the aquariums that are partners in the overall FiSK project. The child can bring up to four family members when visiting the aquarium. The value of the annual pass is 0-275 Dkr per child and 275-365 Dkr per adult depending on the aquarium the child/family chooses to visit (Nordsøen Oceanarium, Kattegatcentret, Fiskeri- og Søfartsmuseet or Den Blå Planet). The family will also receive a small recipe booklet with fish recipes. During the study the child and its family will receive study foods (fish or poultry) free of charge. The provision of free study foods is necessary for executing the study. The value of the gifts and the study foods are not judged to affect the consent for participation.

14. FUNDING

Associate Professor Camilla T. Damsgaard has taken the initiative to the study. The research study FiSK Junior is funded by a 9.0 million DKK grant from Nordea-fonden. A project account will be

created and the account is subject to public audit. The study foods will be bought, possibly with discount, from the food, farming and fisheries industries, mainly from Skagenfood A/S, Sødam via gaardmester.dk, Amanda Seafoods A/S and REMA 1000 Danmark A/S.

The project responsibles have no economic association with the funders, apart from their ordinary employment at NEXS. If other sources of funding are obtained during the study, the project responsible will notify the committee.

15. REFERENCES

1. Fødevarestyrelsen, Ministeriet for Fødevarer, Landbrug og Fiskeri. De officielle kostråd - Spis mere fisk. [Internet]. Available from: http://altomkost.dk/raad-og-anbefalinger/de-officielle-kostraad/spis-mere-fisk/
2. Pedersen AN, Christensen T, Matthiessen J, Kildegaard V, Knudsen M, Rosenlund-Sørensen M, et al. Danskernes kostvaner 2011-2013: Hovedresultater [Internet]. Søborg, Denmark: Technical University of Denmark, National Food Institute, Division of Nutrion; 2015. Available from: www.schultzboghandel.dk
3. Andersen R, Mølgaard C, Skovgaard LT, Brot C, Cashman KD, Chabros E, et al. Teenage girls and elderly women living in northern Europe have low winter vitamin D status. Eur J Clin Nutr. 2005;59(4):533–41.
4. Bucher HC, Hengstler P, Schindler C, Meier G. N-3 polyunsaturated fatty acids in coronary heart disease: a meta-analysis of randomized controlled trials. Am J Med. 2002;112(4):298–304.
5. Lauritzen L, Brambilla P, Mazzocchi A, Harsløf L, Ciappolino V, Agostoni C. DHA Effects in Brain Development and Function. Nutrients. 2016 Jan 4;8(1):6.
6. Hooper L, Harrison RA, Summerbell CD, Moore H, Worthington HV. Omega 3 fatty acids for prevention and treatment of cardiovascular disease. Cochrane Database Syst Rev. 2004;Issue 4:Art. No.: CD003177.
7. Kromhout D. Omega-3 fatty acids and coronary heart disease. The final verdict? Curr Opin Lipidol. 2012;23(6):554–9.
8. Yinko SSLL, Stark KD, Thanassoulis G, Pilote L. Fish Consumption and Acute Coronary Syndrome: A Meta-Analysis. Am J Med. 2014;127(9):848–57.
9. Miller PE, Van Elswyk M, Alexander DD. Long-Chain Omega-3 Fatty Acids Eicosapentaenoic Acid and Docosahexaenoic Acid and Blood Pressure: A Meta-Analysis of Randomized Controlled Trials. Am J Hypertens. 2014;27(7):885–96.
10. Mozaffarian D, Geelen A, Brouwer IA, Geleijnse JM, Zock PL, Katan MB. Effect of Fish Oil on Heart Rate in Humans: A Meta-Analysis of Randomized Controlled Trials. Circulation. 2005;112(13):1945–52.
11. Harris WS. Dietary fish oil and blood lipids. Curr Opin Lipidol. 1996;7:3–7.
12. Lauritzen L, Christensen JH, Damsgaard CT, Michaelsen KF. The effect of fish oil supplementation on heart rate in healthy Danish infants. Pediatr Res. 2008;64(6):610–4.

13. Harsløf LBS, Damsgaard CT, Hellgren LI, Andersen AD, Vogel U, Lauritzen L. Effects on metabolic markers are modified by PPARG2 and COX2 polymorphisms in infants randomized to fish oil. Genes Nutr. 2014;9:396.

14. Damsgaard CT, Schack-Nielsen L, Michaelsen KF, Fruekilde M-B, Hels O, Lauritzen L. Fish oil affects blood pressure and the plasma lipid profile in healthy Danish infants. J Nutr. 2006;136:94–9.

15. Pedersen MH, Mølgaard C, Hellgren LI, Lauritzen L. Effects of fish oil supplementation on markers of the metabolic syndrome. J Pediatr. 2010;157(3):395–400.

16. Moore CS, Bryant SP, Mishra GD, Krebs JD, Browning LM, Miller GJ, et al. Oily fish reduces plasma triacylglycerols: a primary prevention study in overweight men and women. Nutrition. 2006;22(10):1012–24.

17. Hallund J, Overgaard Madsen B, Bügel SH, Jacobsen C, Jakobsen J, Krarup H, et al. The effect of farmed trout on cardiovascular risk markers in healthy men. Br J Nutr. 2010;104(10):1528–36.

18. Rajaram S, Haddad EH, Mejia A, Sabate J. Walnuts and fatty fish influence different serum lipid fractions in normal to mildly hyperlipidemic individuals: a randomized controlled study. Am J Clin Nutr. 2009;89:1657S – 63S.

19. Ramel A, Martinez JA, Kiely M, Bandarra NM, Thorsdottir I. Moderate consumption of fatty fish reduces diastolic blood pressure in overweight and obese European young adults during energy restriction. Nutrition. 2010 Feb;26(2):168–74.

20. Erkkilä AT, Schwab US, de Mello VDF, Lappalainen T, Mussalo H, Lehto S, et al. Effects of fatty and lean fish intake on blood pressure in subjects with coronary heart disease using multiple medications. Eur J Nutr. 2008;47(6):319–28.

21. Erkkilä AT, Schwab US, Lehto S, de Mello VD, Kangas AJ, Soininen P, et al. Effect of fatty and lean fish intake on lipoprotein subclasses in subjects with coronary heart disease: A controlled trial. J Clin Lipidol. 2014;8(1):126–33.

22. Telle-Hansen VH, Larsen LN, Høstmark AT, Molin M, Dahl L, Almendingen K, et al. Daily Intake of Cod or Salmon for 2 Weeks Decreases the 18:1n-9/18:0 Ratio and Serum Triacylglycerols in Healthy Subjects. Lipids. 2012;47(2):151–60.

23. Damsgaard CT, Dalskov S, Laursen RP, Ritz C, Hjorth MF, Lauritzen L, et al. Provision of healthy school meals does not affect the metabolic syndrome score in 8-11-year-old children, but reduces cardiometabolic risk markers despite increasing waist circumference. Br J Nutr. 2014;112(11):1826–36.

24. Damsgaard CT, Ritz C, Dalskov S-M, Landberg R, Stark KD, Biltoft-Jensen A, et al. Associations between school meal-induced dietary changes and metabolic syndrome markers in 8-11-year-old Danish children. Eur J Nutr. In Press.

25. WD Johnson, Kroon J, Greenway F, Bouchard C, Ryan D, Katzmarzyk P. Prevalence of risk factors for metabolic syndrome in adolescents: National Health and Nutrition Examination Survey (NHANES), 2001-2006. Arch Pediatr Adolesc Med. 2009;163:371–7.

26. Webber LS, Srinivasan SR, Wattigney WA, Berenson GS. Tracking of serum lipids and lipoproteins from childhood to adulthood. The Bogalusa Heart Study. Am J Epidemiol. 1991;133(9):884–99.

27. Juhola J, Magnussen CG, Viikari JSA, Kähönen M, Hutri-Kähönen N, Jula A, et al. Tracking of Serum Lipid Levels, Blood Pressure, and Body Mass Index from Childhood to Adulthood: The Cardiovascular Risk in Young Finns Study. J Pediatr. 2011;159(4):584–90.

28. Lauritzen L, Hansen HS, Jørgensen MH, Michaelsen KF. The essentiality of long chain n-3 fatty acids in relation to development and function of the brain and retina. Prog Lipid Res. 2001;(40):1–94.

29. Simmer K, Schulzke S, Patole S. Longchain polyunsaturated fatty acid supplementation in preterm infants. Cochrane Database Syst Rev. 2008;Issue 1:Art. No.: CD000375.

30. Simmer K, Patole S, Rao SC. Longchain polyunsaturated fatty acid supplementation in infants born at term. Cochrane Database Syst Rev. 2008;Issue 1:Art No.: CD000376.

31. Qawasmi A, Landeros-Weisenberger A, Leckman JF, Bloch MH. Meta-analysis of Long-Chain Polyunsaturated Fatty Acid Supplementation of Formula and Infant Cognition. Pediatrics. 2012;129(6):1141–9.

32. Makrides M, Gibson RA, McPhee AJ, Yelland L, Quinlivan J, Ryan P, et al. Effect of DHA supplementation during pregnancy on maternal depression and neurodevelopment of young children: a randomized controlled trial. Jama. 2010;304(15):1675–83.

33. Lauritzen L, Jorgensen MH, Olsen SF, Straarup EM, Michaelsen KF. Maternal fish oil supplementation in lactation: effect on developmental outcome in breast-fed infants. Reprod Nutr Dev. 2005 Sep;45(5):535–47.

34. Delgado-Noguera MF, Bonfill Cosp X. Supplementation with long chain polyunsaturated fatty acids (LCPUFA) to breastfeeding mothers for improving child growth and development. Cochrane Database of Systematic Reviews. 2010;Issue 12. Art. No.: CD007901:DOI: 10.1002/14651858.CD007901.pub2.

35. Jiao J, Li Q, Chu J, Zeng W, Yang M, Zhu S. Effect of n-3 PUFA supplementation on cognitive function throughout the life span from infancy to old age: a systematic review and meta-analysis of randomized controlled trials. Am J Clin Nutr. 2014;100:1422–36.

36. Dalton A, Wolmarans P, Witthuhn RC, van Stuijvenberg ME, Swanevelder SA, Smuts CM. A randomised control trial in schoolchildren showed improvement in cognitive function after consuming a bread spread, containing fish flour from a marine source. Prostaglandins Leukot Essent Fatty Acids. 2009;80:143–9.

37. Parletta N, Cooper P, Gent DN, Petkov J, O'Dea K. Effects of fish oil supplementation on learning and behaviour of children from Australian Indigenous remote community schools: A randomised controlled trial. Prostaglandins Leukot Essent Fat Acids PLEFA. 2013 Aug;89(2-3):71–9.

38. Baumgartner J, Smuts CM, Malan L, Kvalsvig J, van Stuijvenberg ME, Hurrell RF, et al. Effects of iron and n-3 fatty acid supplementation, alone and in combination, on cognition in school children: a randomized, double-blind, placebo-controlled intervention in South Africa. Am J Clin Nutr. 2012;96:1327–38.

39. Osendarp SJM, Baghurst KK, Bryan J, Calvaresi E, Hughes D, Hussaini M, et al. Effect of a 12-mo micronutrient intervention on learning and memory in well-nourished and marginally nourished school-aged children: 2 parallel, randomized, placebo-controlled studies in Australia and Indonesia. Am J Clin Nutr. 2007;86:1082–93.

40. Kennedy DO, Jackson PA, Elliot JM, Scholey AB, Robertson BC, Greer J, et al. Cognitive and mood effects of 8 weeks' supplementation with 400 mg or 1000 mg of the omega-3 essential fatty acid docosahexaenoic acid (DHA) in healthy children aged 10–12 years. Nutr Neurosci. 2009;12(2):48–56.

41. McNamara RK, Carlson SE. Role of omega-3 fatty acids in brain development and function: Potential implications for the pathogenesis and prevention of psychopathology. Prostaglandins Leukot Essent Fatty Acids. 2006;75(4-5):329–49.

42. Lavialle M, Denis I, Guesnet P, Vancassel S. Involvement of omega-3 fatty acids in emotional responses and hyperactive symptoms. J Nutr Biochem. 2010;21(10):899–905.

43. Hennebelle M, Champeil-Potokar G, Lavialle M, Vancassel S, Denis I. Omega-3 polyunsaturated fatty acids and chronic stress-induced modulations of glutamatergic neurotransmission in the hippocampus. Nutr Rev. 2014;72(2):99–112.

44. Kirby A, Woodward A, Jackson S, Wang Y, Crawford MA. A double-blind, placebo-controlled study investigating the effects of omega-3 supplementation in children aged 8–10 years from a mainstream school population. Res Dev Disabil. 2010;31(3):718–30.

45. Smuts CM, Greeff J, Kvalsvig J, Zimmermann MB, Baumgartner J. Long-chain n-3 PUFA supplementation decreases physical activity during class time in iron-deficient South African school children. Br J Nutr. 2015;113:212–24.

46. Weissman MM, Bland RC, Canino GJ, Faravelli C, Greenwald S, Hwu H-G, et al. Cross-National Epidemiology of Major Depression and Bipolar Disorder. JAMA. 1996;276(4):293–9.

47. Lin P-Y, Huang S-Y, Su K-P. A Meta-Analytic Review of Polyunsaturated Fatty Acid Compositions in Patients with Depression. Biol Psychiatry. 2010;68(2):140–7.

48. McNamara RK, Vannest JJ, Valentine CJ. Role of perinatal long-chain omega-3 fatty acids in cortical circuit maturation: Mechanisms and implications for psychopathology. World J Psychiatry. 2015;5(1):15–34.

49. Ygil KH. Mål, vægt og portionsstørrelser på fødevarer [Internet]. Søborg, Denmark: Technical University of Denmark, National Food Institute, Division of Nutrition; 2013. Available from: www.food.dtu.dk

50. Møller Christensen L, Kørup K, Trolle E, Matthiessen J, Fagt S. Børn og unges måltidsvaner 2005-2008. National Food Institute, Technical University of Denmark, Division of Nutrition; 2012.

51. World Cancer Research Fund/American Institute for Cancer Research. Food, nutrition, physical activity and the prevention of cancer: a global perspective. Washington, D.C; 2007.

52. Sørensen LB, Damsgaard CT, Dalskov S-M, Petersen RA, Egelund N, Dyssegaard CB, et al. Diet-induced changes in iron and n-3 fatty acid status and associations with cognitive performance in 8–11-year-old Danish children: secondary analyses of the Optimal Well-Being, Development and Health for Danish Children through a Healthy New Nordic Diet School Meal Study. Br J Nutr. 2015;114:1623–37.

53. Rita Hannisdal, Nøstbakken OJ, Lunestad BT, Hove HT, Graff IE, Madsen L. Monitoring program for pharmaceuticals, illegal substances, and contaminants in farmed fish. Commissioned by the Norwegian Food Safety Authority: National Institute of Nutrition and Seafood Research (NIFES); 2015 p. 34.

54. VKM. Benefit-risk assessment of fish and fish products in the Norwegian diet - an update [Internet]. Oslo, Norway: Scientific Opinion of the Scientific Steering Committee; 2014 p. 293. Report No.: 15. Available from: www.vkm.no

55. European Food Safety Authority. EFSA Panel on Contaminants in the Food Chain (CONTAM); Scientific Opinion on the risk for public health related to the presence of mercury and methylmercury in food. EFSA Journal. 2012;10(12):2985[241pp.].

56. European Commission, Scientific Committee on Food. Opinion of the Scientific Committe on Food on the risk assessment of dioxins and dioxin-like PBSs in food. Bruxelles, Belgium; 2001.

57. Piger, Højde og vægt for alder, fødsel til 20 år [Internet]. Vækst og Reproduktion, Rigshospitalet. 2014. Available from: http://www.vækstkurver.dk/pige.html

58. Drenge, Højde og vægt for alder, fødsel til 20 år [Internet]. Vækst og Reproduktion, Rigshospitalet; 2014. Available from: http://www.vækstkurver.dk/dreng.html

59. Petersen A, Fromberg A, Andersen JH, Sloth JJ, Granby K, Duedahl-Olesen L, et al. Chemical contaminants 2004-2011. Kgs. Lyngby: National Food Institute, Technical University of Denmark; 2013.

60. Birnie KA, Noel M, Chambers CT, von Baeyer CL, Fernandez CV. The Cold Pressor Task: Is it an Ethically Acceptable Pain Research Method in Children? J Pediatr Psychol. 2011 Nov 1;36(10):1071–81.

61. von Baeyer CL, Piira T, Chambers C, Trapanotto M, Zeltzer L. Guidelines for the cold pressor task as an experimental pain stimulus for use with children. J Pain. 2005 Apr;6(4):218–27.

62. The Helsinki Declaration - updated at the 64th World Medical Association General Assembly. Fortaleza, Brazil; 2013.

16. APPENDICES (all in Danish)

1. Protocol resume [Protokolresumé]
2. Invitation letter with response form [Invitationsbrev med interesseblanket]
3. Participant information material [Deltagerinformation]
4. Consent forms [Samtykkeerklæringer]
    a. Child [Barn]
    b. Biobank [Barn]
5. Parental empowerment proxy form [Forældrefuldmagt]
6. Recruitment advertisement [Rekrutteringsannonce]
7. Lifestyle questionnaire – baseline visit [Spørgeskema om solvaner og husstand]
8. Lifestyle questionnaire – endpoint visit [Spørgeskema om solvaner]
9. Behaviour Rating Inventory of Executive Function questionnaire (BRIEF) for parents [BRIEF - Adfærdsvurdering af eksekutiv funktion]
10. Strengths and Difficulties Questionnaires (SDQ) for parents [Spørgeskema om styrker og vanskeligheder]
11. Quality of Life Questionnaire (KINDL) for parents [Spørgeskema for livskvalitet for børn og unge (KINDL)]
12. d2-test – Classic test of attention and concentration [d2-testen - Klassisk test af opmærksomhed og koncentration]
13. Pubertal stage questionnaire (girls) [Spørgeskema om pubertet - piger]
14. Pubertal stage questionnaire (boys) [Spørgeskema om pubertet - drenge]
15. Instructions on recording of 4 days dietary recording [Vejledning i 4 dages kostregistrering]
16.
17. Instructions on recording of physical activity by accelerometer [Instruktion i fysisk aktivitetsmåling]
18. Instructions on fasting and use of EMLA patches [Vejledning i faste og brug af EMLA plaster]
19. Food frequency questionnaire [Spørgeskema Fiske- og kødindtag]
20. Quality of Life Questionnaire (Kid-KINDL) for children [Spørgeskema for livskvalitet for børn (Kid-KINDL)]
21. Adverse events and reactions form [Registrering af hændelser]
