# Supplementary figures and images for: Exploring correlations between neuropsychological measures and domain-specific consistency in associations with n-3 LCPUFA status in 8-9 year-old boys and girls
Source: PLoS One. 2019 May 22;14(5):e0216696. doi: 10.1371/journal.pone.0216696 (PMC6530844; doi:10.1371/journal.pone.0216696)

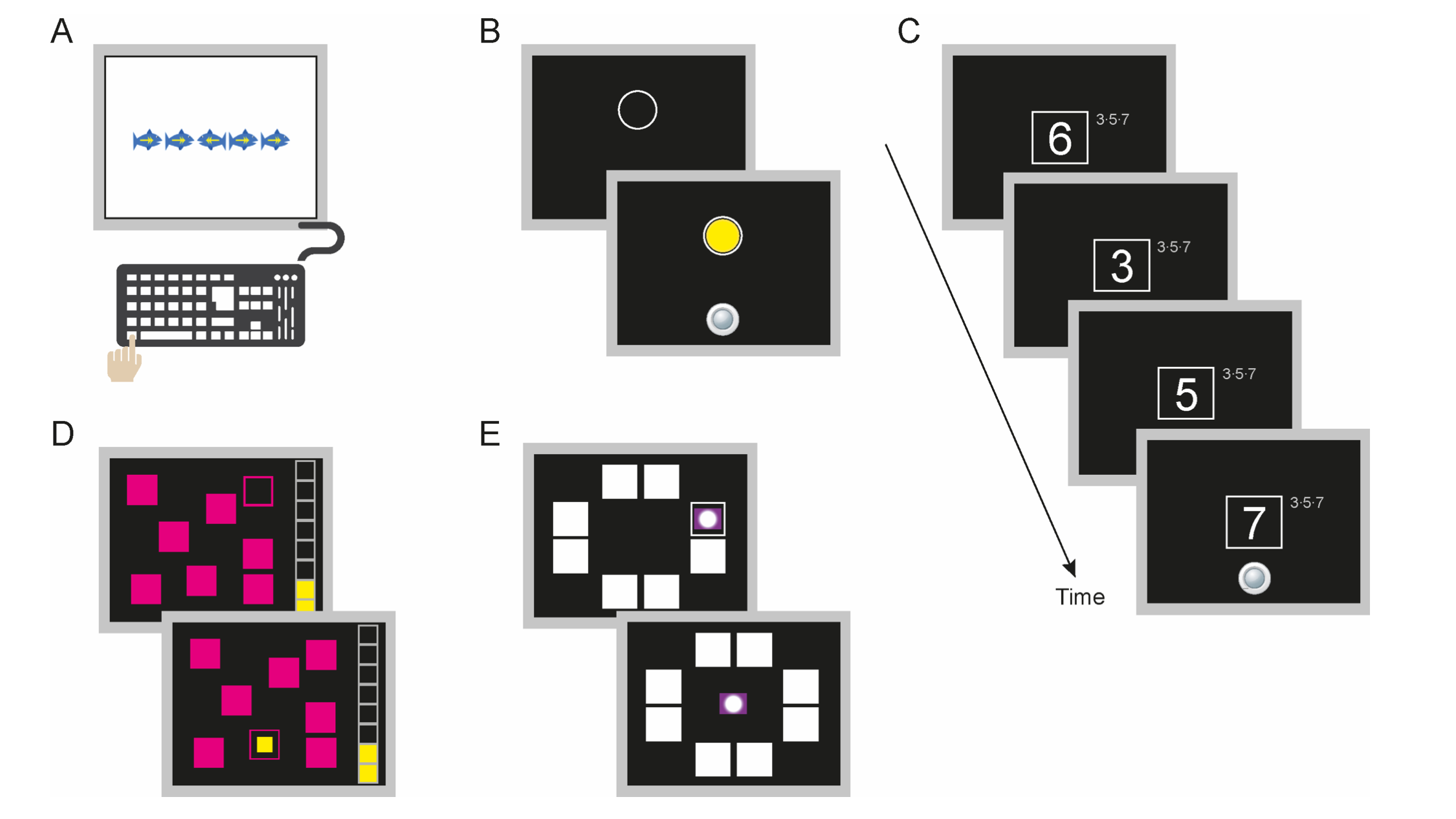

Supplement: S1 Fig — A. Flanker task. B. Reaction time (RTI). C. Rapid Visual Processing (RVP). D. Spatial Working Memory (SWM). E. Paired Associates Learning (PAL). (TIFF) [file pone.0216696.s002.tiff]

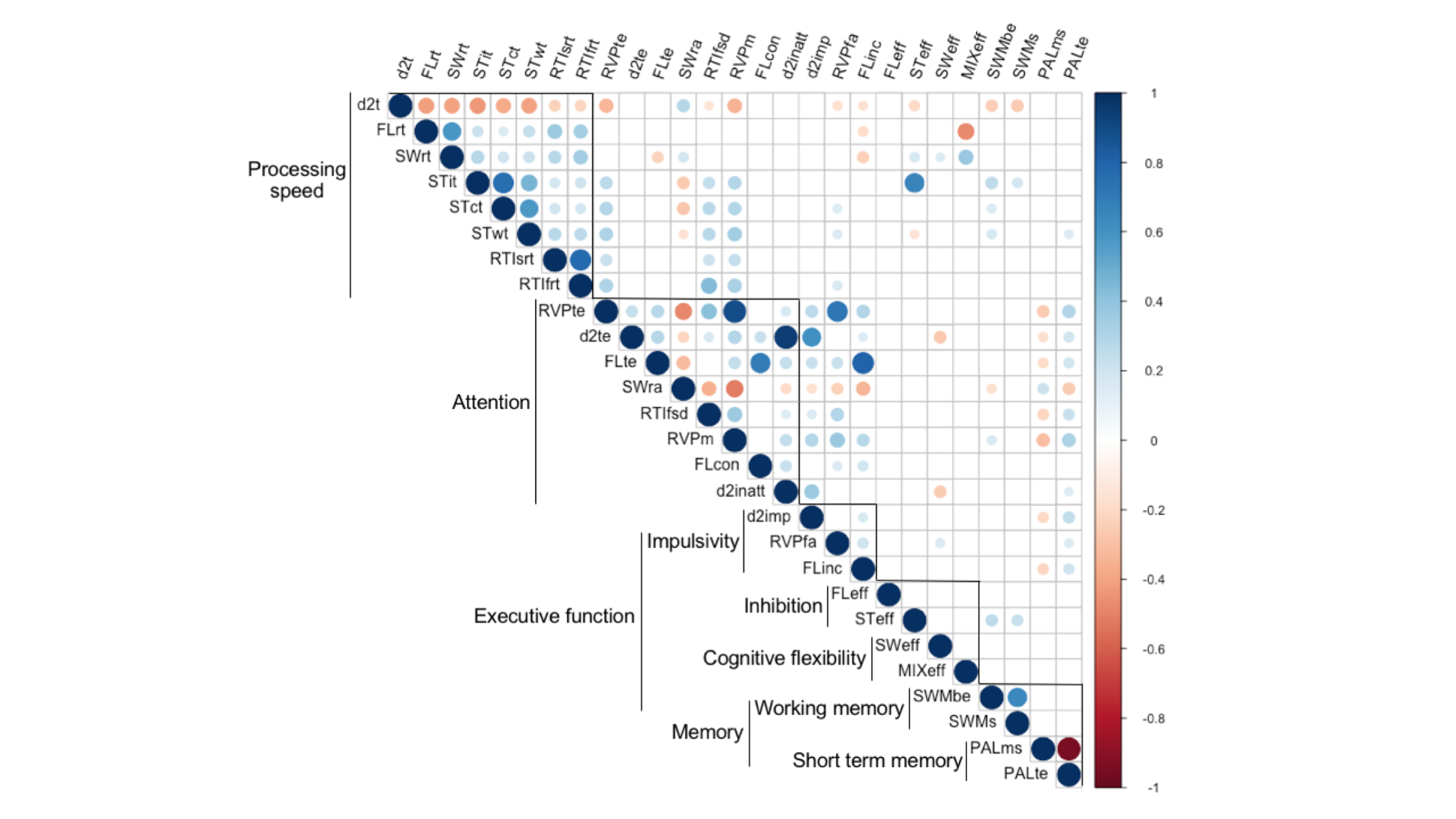

Supplement: S2 Fig — Blue circles show positive correlations, red circles show negative correlations. Color intensity and size of the circle indicates strength of the association. Blank fields indicate insignificant (p>0.05) correlation. SWra, switch task response accuracy %; PALms, Paired associates learning memory score; d2t, d2 Processing speed; MIXeff, mixing cost; SWeff, switch effect; FLeff, Flanker effect; STeff, Stroop effect; SWrt, switch task reaction time; STit, Stroop color-word time; STct, Stroop color time; STwt, Stroop word time; RTIsrt, simple reaction time; RTIfrt, five-choice reaction time; FLrt, Flanker reaction time; SWMbe, SWM total between errors; SWMs, SWM strategy; FLcon, Flanker congruent error%; FLinc, Flanker incongruent error%; FLte, Flanker total error%; RVPte, RVP total error%; RVPm, RVP misses%; RVPfa, RVP false alarm%; PALte, PAL total error%; d2te, d2 total error%; d2inatt, d2 inattention error%; d2imp, d2 impulsivity error%; RTIfsd, five-choice reaction time SD. (TIFF) [file pone.0216696.s003.tiff]

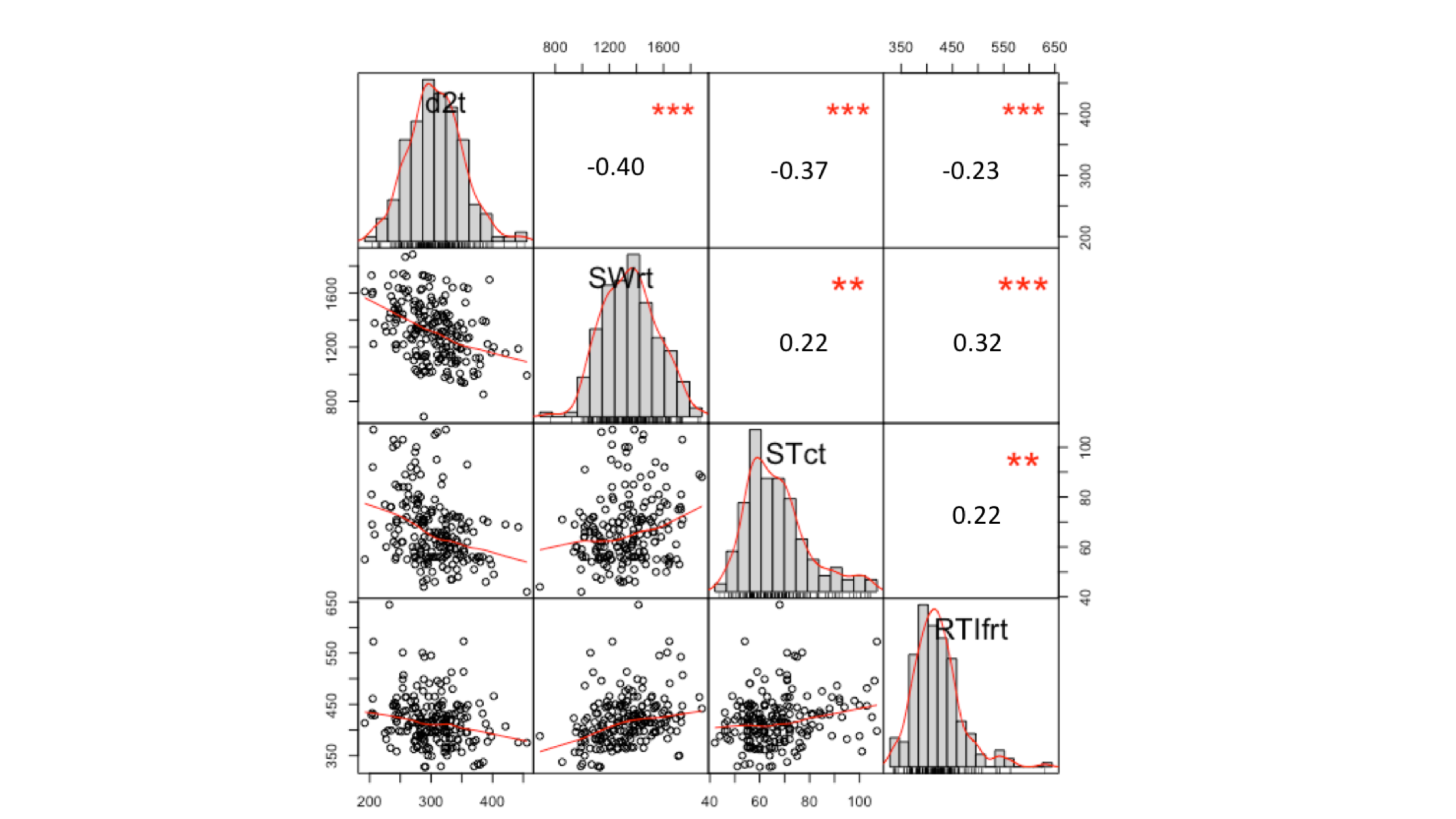

Supplement: S3 Fig — Numbers indicate Pearson correlations. *p<0.05, **p<0.01, ***p<0.001. d2t, d2 Processing speed; SWrt, switch task reaction time; STct, Stroop color time; RTIfrt, Reaction time five-choice reaction time. (TIFF) [file pone.0216696.s004.tiff]

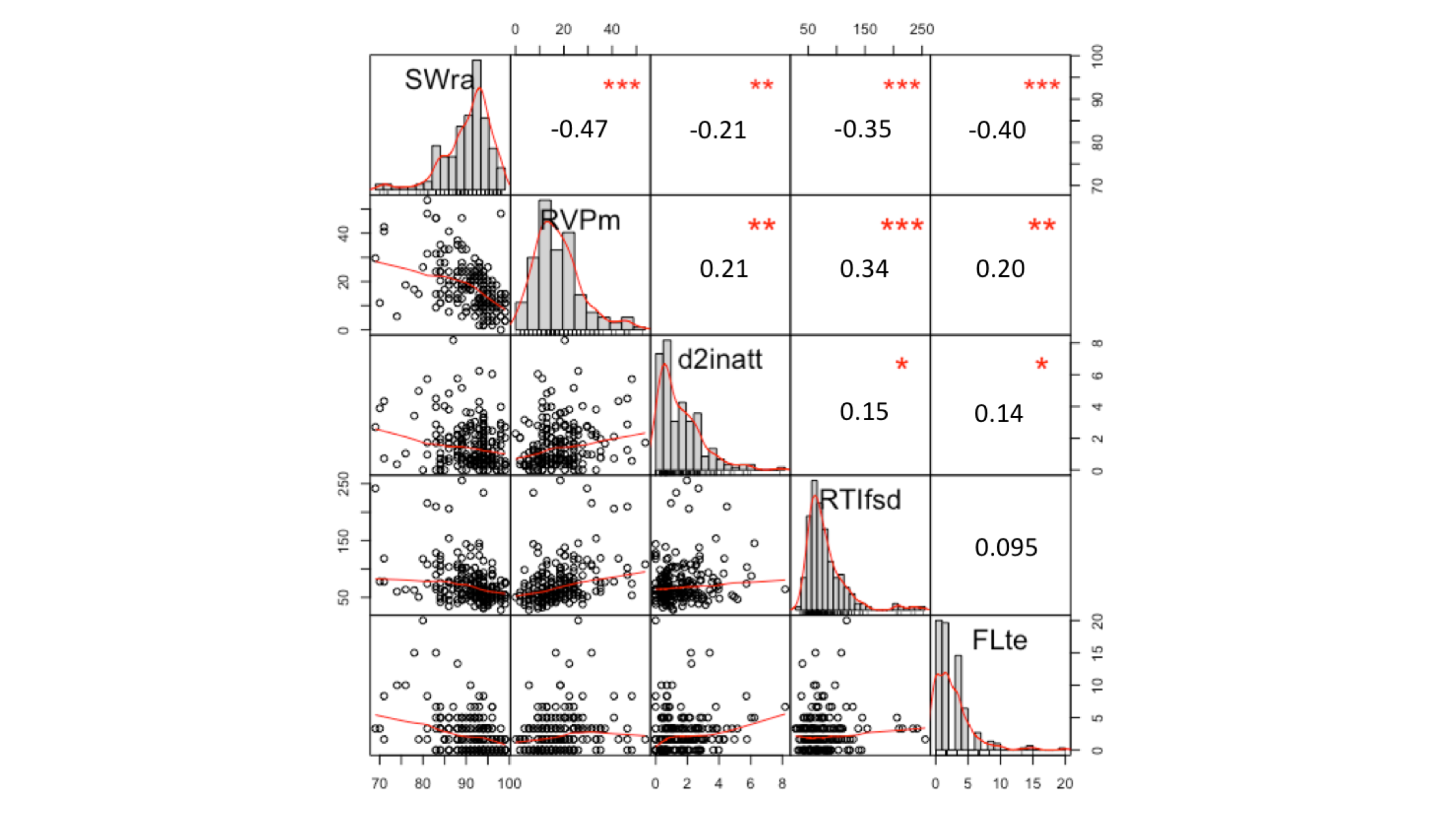

Supplement: S4 Fig — Numbers indicate Spearman correlations. *p<0.05, **p<0.01, ***p<0.001. SWra, switch task response accuracy %; RVPm, RVP misses %; d2inatt, d2 inattention error%; RTIfsd, Reaction time five-choice reaction time SD; FLte, Flanker total error %. One d2inatt outlier (>15) and two RTIfsd outliers (>300) removed from the analysis. (TIFF) [file pone.0216696.s005.tiff]

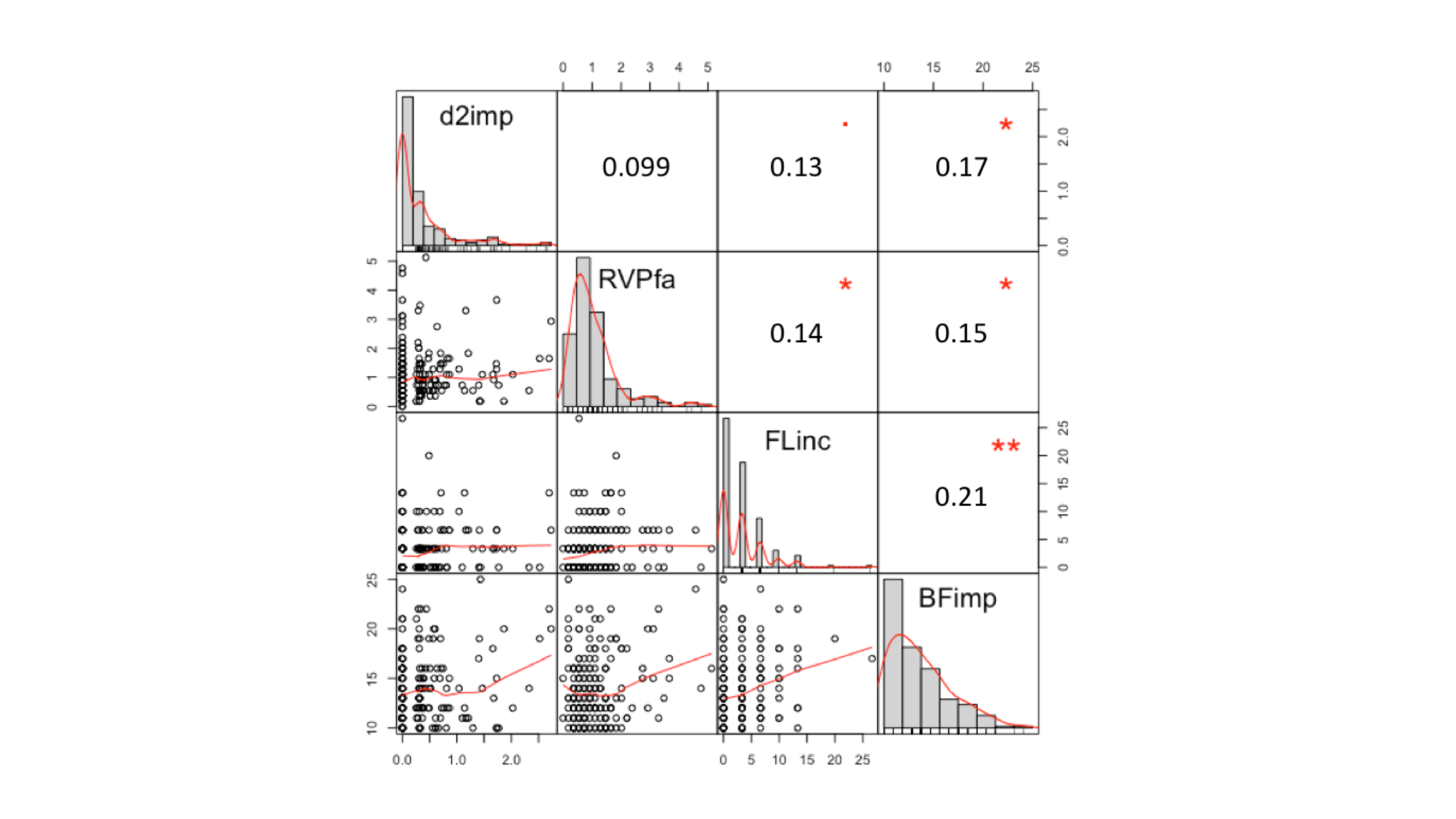

Supplement: S5 Fig — Numbers indicate Spearman correlations. •p<0.1, *p<0.05, **p<0.01. d2imp, d2 impulsivity error%; RVPfa, RVP false alarm%; FLinc, Flanker incongruent error %; BFimp, BRIEF impulsivity. (TIFF) [file pone.0216696.s006.tiff]

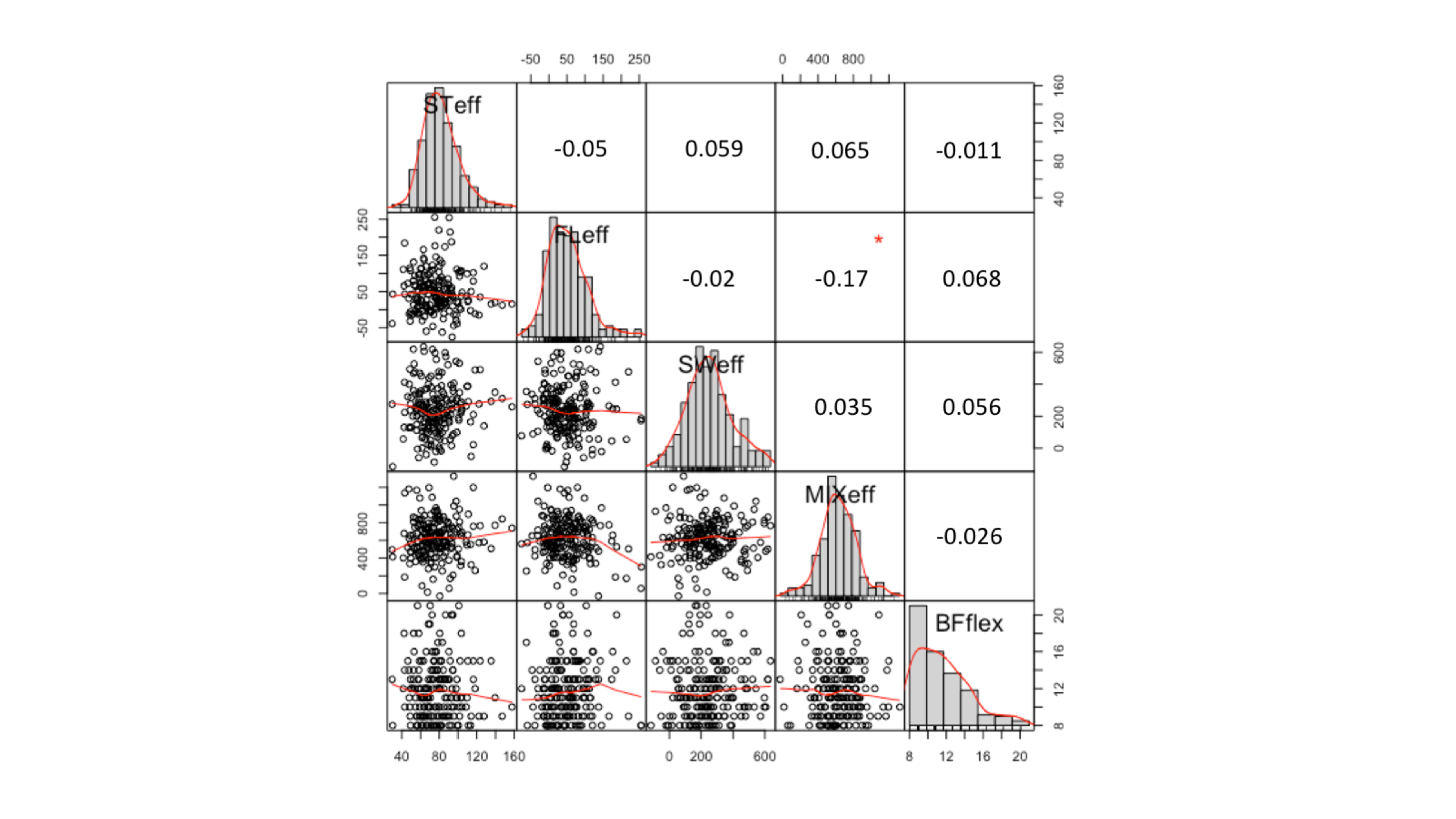

Supplement: S6 Fig — Numbers indicate Spearman correlations. *p<0.05. STeff, Stroop effect; FLeff, Flanker effect; SWeff, switch cost; MIXeff, mixing cost; BFflex, BRIEF flexibility. (TIFF) [file pone.0216696.s007.tiff]

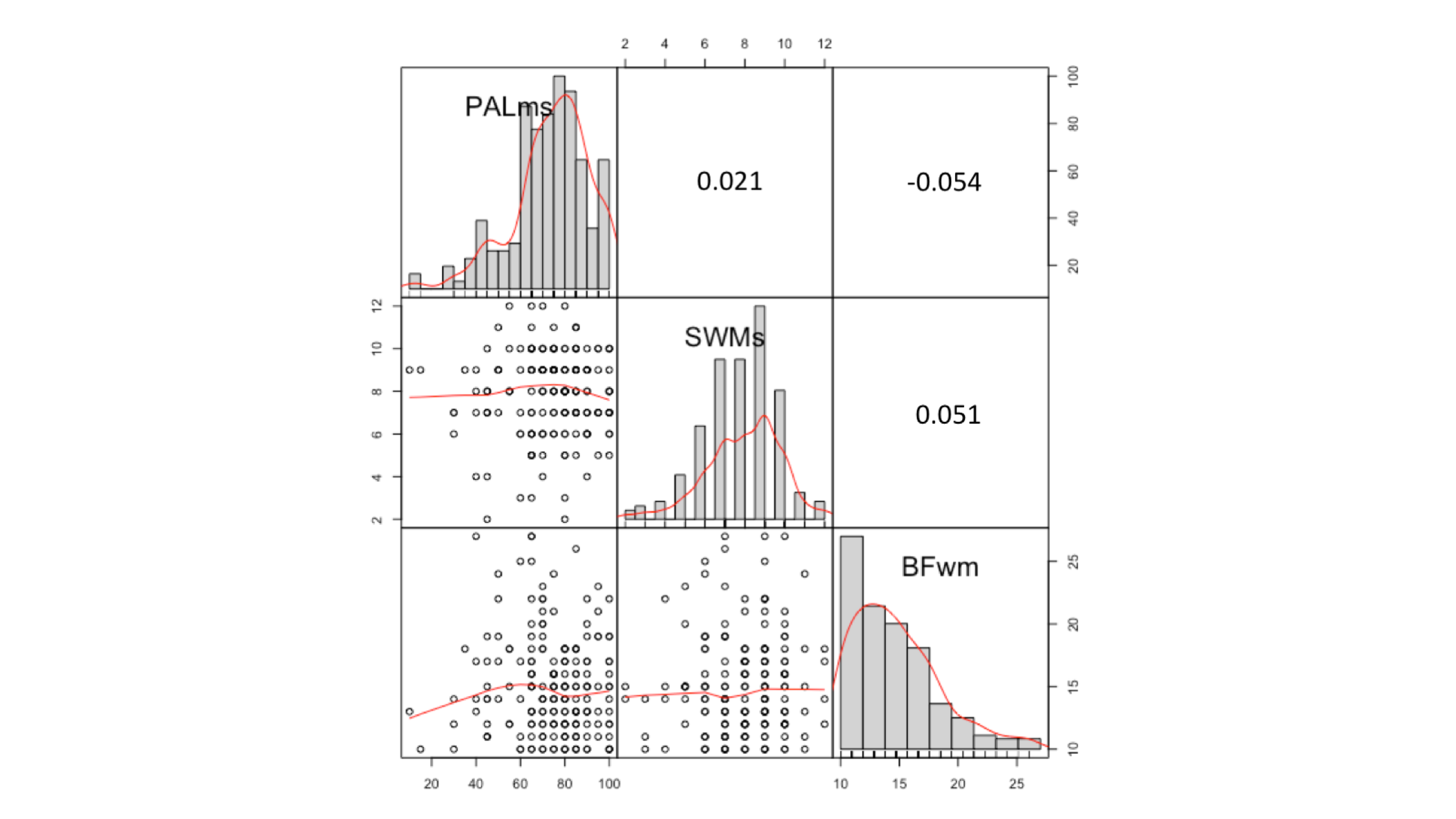

Supplement: S7 Fig — Numbers indicate Spearman correlations. *p<0.05, **p<0.01, ***p<0.001. PALms, Paired associates learning memory score; SWMs, SWM strategy; BFwm, BRIEF working memory. (TIFF) [file pone.0216696.s008.tiff]

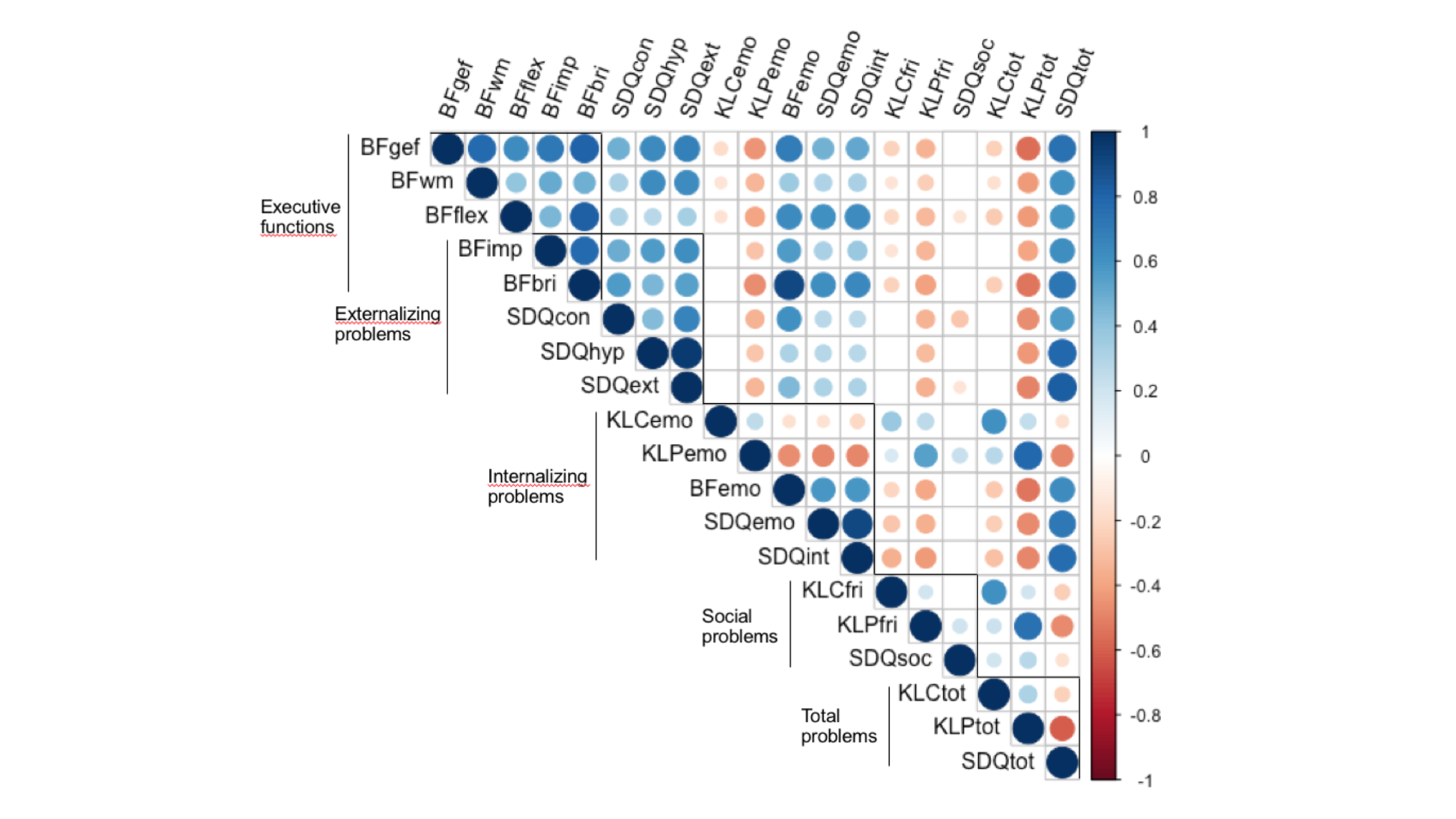

Supplement: S8 Fig — Blue circles show positive correlations, red circles show negative correlations. Color intensity and size of the circle indicates strength of the association. Blank fields indicate insignificant (p>0.05) correlation. BFgef, BRIEF general executive function; BFwm, BRIEF working memory; BFflex, BRIEF flexibility; BFimp, BRIEF impulsivity; BFbri, BRIEF behavior regulation index; SDQcon, SDQ conduct problems; SDQhyp, SDQ hyperactivity/inattention; SDQext, SDQ externalizing problems; KLCemo, KINDL child emotional well-being; KLPemo, KINDL parent emotional well-being; BFemo, BRIEF emotional control; SDQemo, SDQ emotional symptoms; SDQint, SDQ internalizing problems; KLCfri, KINDL child friends; KLPfri, KINDL parent friends; SDQsoc, SDQ prosocial behavior; KLCtot, KINDL child total well-being; KLPtot, KINDL parent total well-being; SDQtot, SDQ total difficulties. (TIFF) [file pone.0216696.s009.tiff]

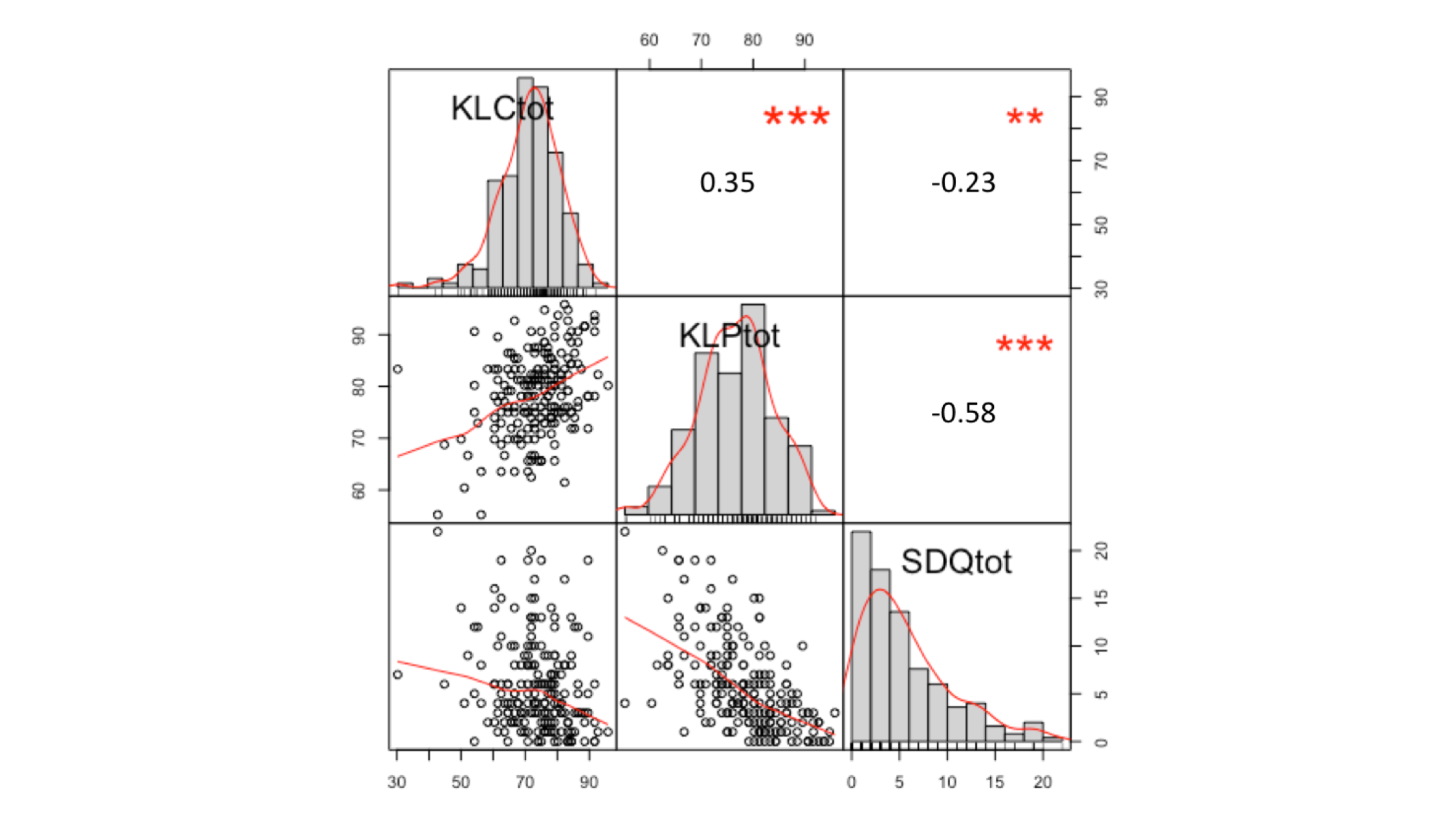

Supplement: S9 Fig — Numbers indicate Spearman correlations. **p<0.01, ***p<0.001.KLCtot, KINDL child total well-being; KLPtot, KINDL parent total well-being; SDQtot, SDQ total difficulties. (TIFF) [file pone.0216696.s010.tiff]

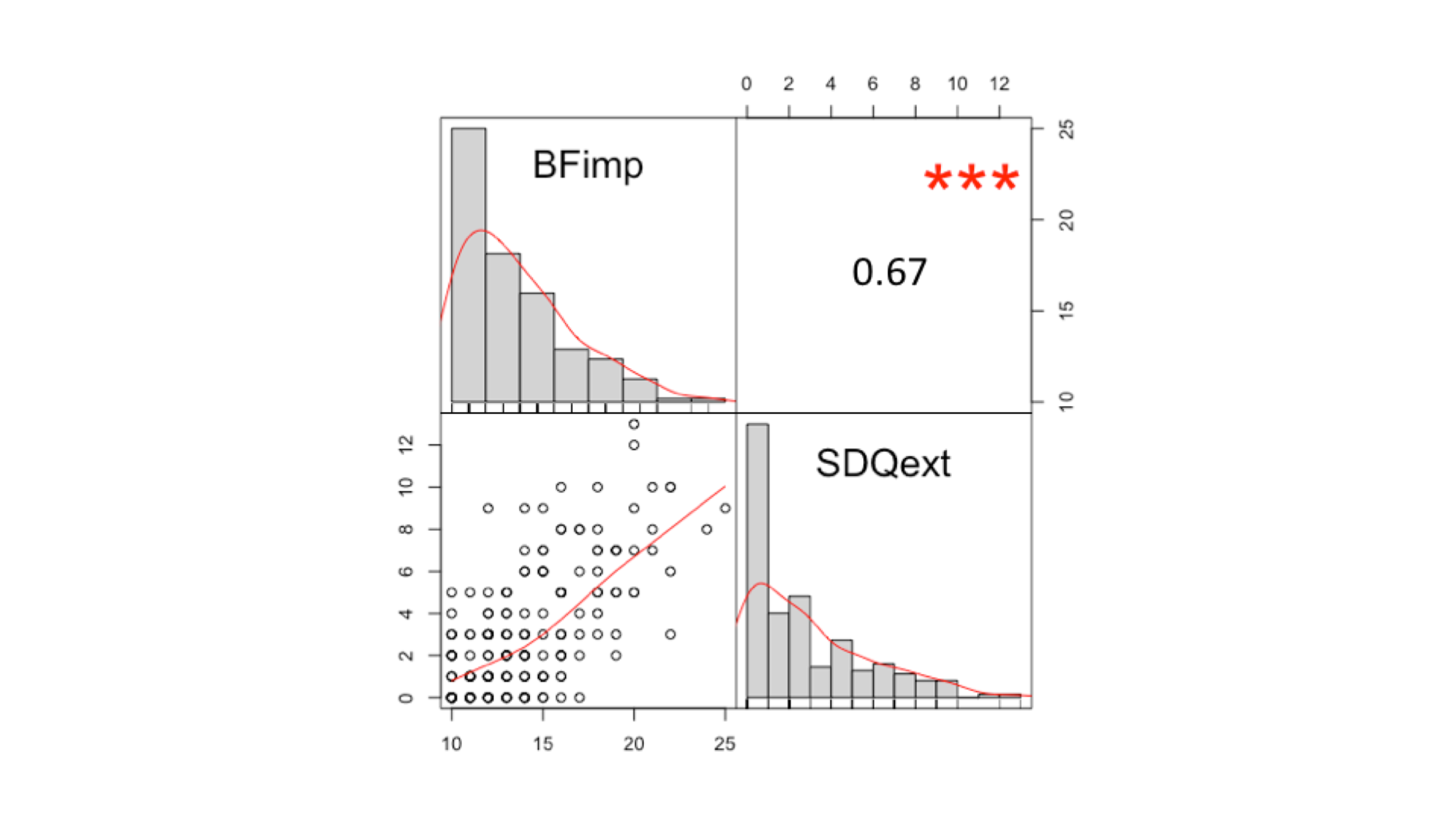

Supplement: S10 Fig — Numbers indicate Spearman correlations. ***p<0.001. BFimp, BRIEF impulsivity; SDQext, SDQ externalizing problems. (TIFF) [file pone.0216696.s011.tiff]

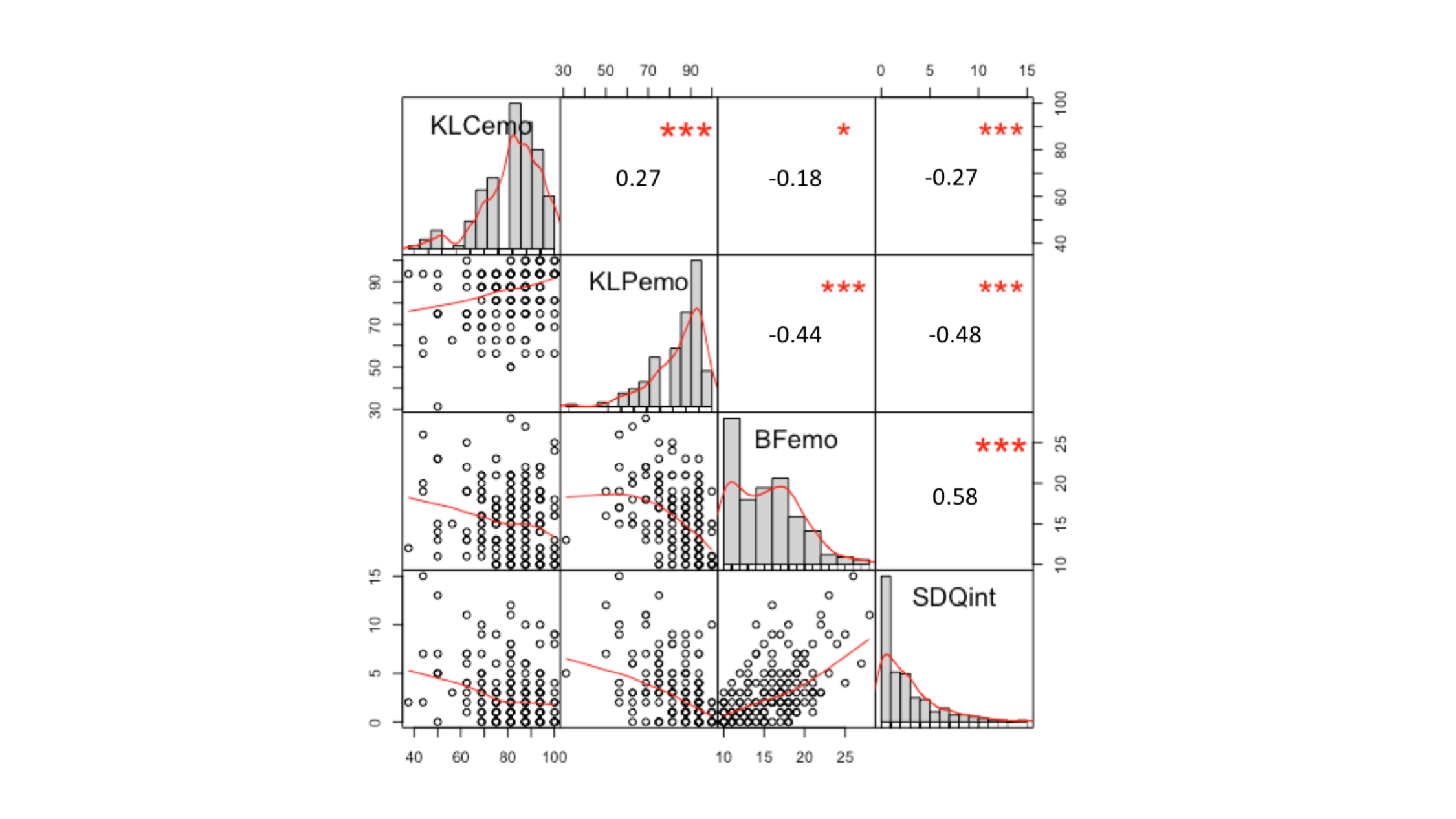

Supplement: S11 Fig — Numbers indicate Spearman correlations. *p<0.05, **p<0.01, ***p<0.001. KLCemo, KINDL child emotional well-being; KLPemo, KINDL parent emotional well-being; BFemo, BRIEF emotional control; SDQint, SDQ internalizing problems. (TIFF) [file pone.0216696.s012.tiff]

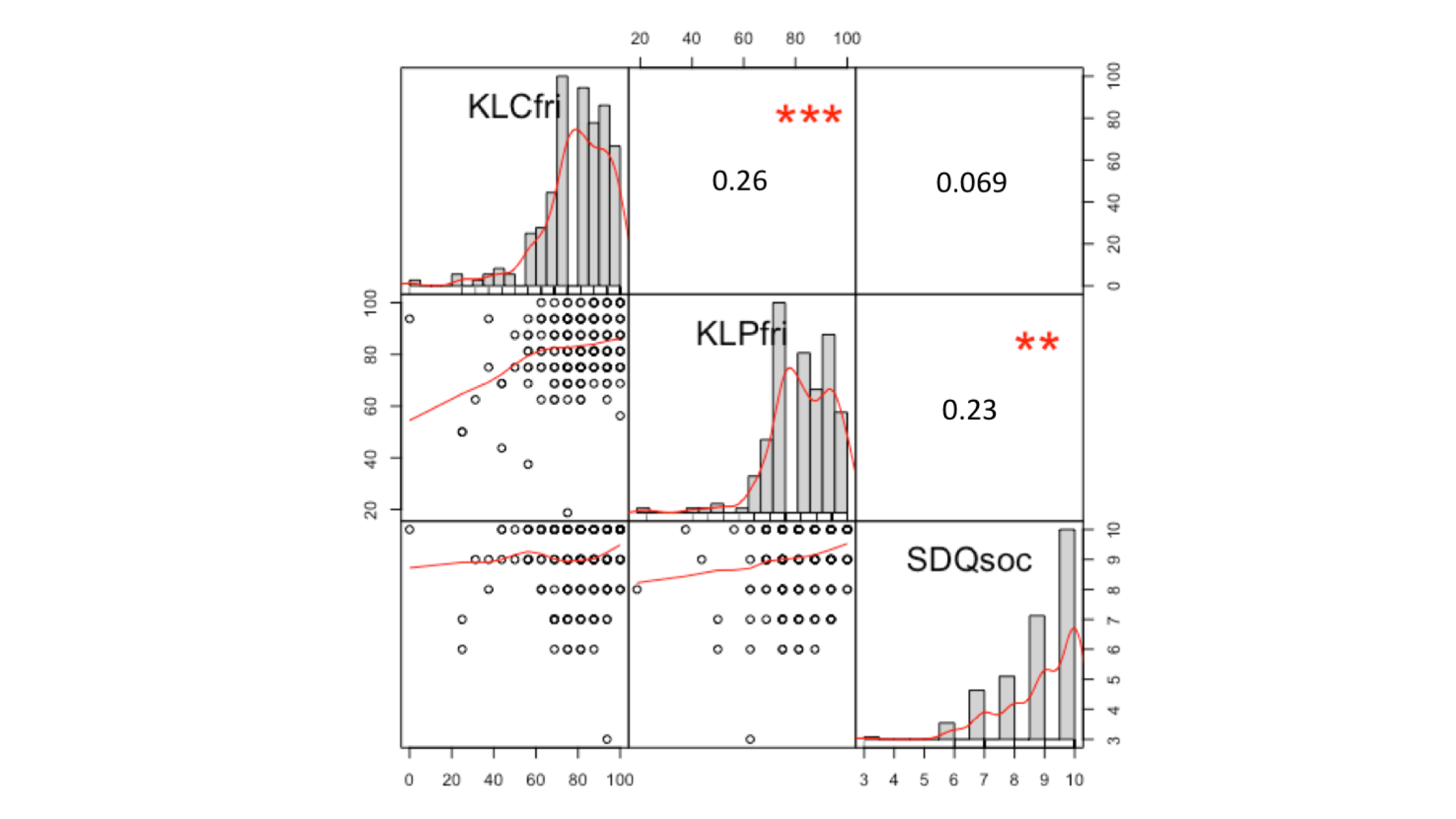

Supplement: S12 Fig — Numbers indicate Spearman correlations. **p<0.01, ***p<0.001. KLCfri, KINDL child friends; KLPfri, KINDL parent friends; SDQsoc, SDQ prosocial behavior. (TIFF) [file pone.0216696.s013.tiff]
